# Lifestyle and dietary interventions for Ménière’s disease

**DOI:** 10.1002/14651858.CD015244.pub2

**Published:** 2023-02-27

**Authors:** Katie E Webster, Ben George, Ambrose Lee, Kevin Galbraith, Natasha A Harrington-Benton, Owen Judd, Diego Kaski, Otto R Maarsingh, Samuel MacKeith, Louisa Murdin, Jaydip Ray, Vincent A Van Vugt, Martin J Burton

**Affiliations:** Cochrane ENT, Nuffield Department of Surgical SciencesUniversity of OxfordOxfordUK; Corpus Christi CollegeUniversity of OxfordOxfordUK; Department of Otolaryngology - Head and Neck SurgeryUniversity of TorontoTorontoCanada; Cochrane ENTNuffield Department of Surgical Sciences, University of OxfordOxfordUK; Ménière’s SocietyWootonUK; ENT DepartmentUniversity Hospitals of Derby and Burton NHS Foundation TrustDerbyUK; National Hospital for Neurology and NeurosurgeryLondonUK; Department of General Practice, Amsterdam UMCVrije Universiteit Amsterdam, Amsterdam Public Health Research InstituteAmsterdamNetherlands; ENT DepartmentOxford University Hospitals NHS Foundation TrustOxfordUK; ENT DepartmentGuy's and St Thomas' NHS Foundation TrustLondonUK; University of SheffieldSheffieldUK; Cochrane UKOxfordUK

**Keywords:** Adult, Humans, Caffeine, Life Style, Meniere Disease, Randomized Controlled Trials as Topic, Sodium Chloride, Tinnitus, Tinnitus/etiology, Tinnitus/prevention & control, Vertigo, Vertigo/etiology, Vertigo/prevention & control

## Abstract

**Background:**

Ménière's disease is a condition that causes recurrent episodes of vertigo, associated with hearing loss and tinnitus. Lifestyle or dietary modifications (including reducing the amount of salt or caffeine in the diet) are sometimes suggested to be of benefit for this condition. The underlying cause of Ménière's disease is unknown, as is the way in which these interventions may work. The efficacy of these different interventions at preventing vertigo attacks, and their associated symptoms, is currently unclear.

**Objectives:**

To evaluate the benefits and harms of lifestyle and dietary interventions versus placebo or no treatment in people with Ménière's disease.

**Search methods:**

The Cochrane ENT Information Specialist searched the Cochrane ENT Register; Central Register of Controlled Trials (CENTRAL); Ovid MEDLINE; Ovid Embase; Web of Science; ClinicalTrials.gov; ICTRP and additional sources for published and unpublished trials. The date of the search was 14 September 2022.

**Selection criteria:**

We included randomised controlled trials (RCTs) and quasi‐RCTs in adults with Ménière's disease comparing any lifestyle or dietary intervention with either placebo or no treatment. We excluded studies with follow‐up of less than three months, or with a cross‐over design (unless data from the first phase of the study could be identified).

**Data collection and analysis:**

We used standard Cochrane methods. Our primary outcomes were: 1) improvement in vertigo (assessed as a dichotomous outcome ‐ improved or not improved), 2) change in vertigo (assessed as a continuous outcome, with a score on a numerical scale) and 3) serious adverse events. Our secondary outcomes were: 4) disease‐specific health‐related quality of life, 5) change in hearing, 6) change in tinnitus and 7) other adverse effects. We considered outcomes reported at three time points: 3 to < 6 months, 6 to ≤ 12 months and > 12 months. We used GRADE to assess the certainty of evidence for each outcome.

**Main results:**

We included two RCTs, one related to diet, and the other related to fluid intake and sleep. In a Swedish study, 51 participants were randomised to receive 'specially processed cereals' or standard cereals. The specially processed cereals are thought to stimulate the production of anti‐secretory factor ‐ a protein that reduces inflammation and fluid secretion. Participants received the cereals for three months. The only outcome reported by this study was disease‐specific health‐related quality of life.

The second study was conducted in Japan. The participants (223) were randomised to receive abundant water intake (35 mL/kg/day), or to sleep in darkness (in an unlit room for six to seven hours per night), or to receive no intervention. The duration of follow‐up was two years. The outcomes assessed were 'improvement in vertigo' and hearing.

As these studies considered different interventions we were unable to carry out any meta‐analysis, and for almost all outcomes the certainty of the evidence was very low. We are unable to draw meaningful conclusions from the numerical results.

**Authors' conclusions:**

The evidence for lifestyle or dietary interventions for Ménière's disease is very uncertain. We did not identify any placebo‐controlled RCTs for interventions that are frequently recommended for those with Ménière's disease, such as salt restriction or caffeine restriction. We identified only two RCTs that compared a lifestyle or dietary intervention to placebo or no treatment, and the evidence that is currently available from these studies is of low or very low certainty. This means that we have very low confidence that the effects reported are accurate estimates of the true effect of these interventions. Consensus on the appropriate outcomes to measure in studies of Ménière's disease is needed (i.e. a core outcome set) in order to guide future studies in this area and enable meta‐analyses of the results. This must include appropriate consideration of the potential harms of treatment, as well as the benefits.

## Plain language summary

**What are the benefits and risks of different lifestyle and dietary changes for Ménière's disease?**

**Key messages**

Due to a lack of robust evidence, it is not clear whether any lifestyle or dietary changes used to treat Ménière's disease work to improve people's symptoms, despite their routine use in clinical practice. We also do not know if there are any risks associated with these interventions.

Larger, well‐conducted studies are needed in order to identify whether any lifestyle or dietary changes may be effective, and assess whether they have any harmful effects.

Further work also needs to be done to find out how best to measure the symptoms of people with Ménière's disease, in order to assess whether treatments are beneficial or not. This should include the development of a 'core outcome set' ‐ a list of things that should be measured in all studies on Ménière's disease.

**What is Ménière's disease?**

Ménière's disease is a condition that affects the inner ear. It causes repeated attacks of dizziness or vertigo (a spinning sensation), together with hearing problems, tinnitus (ringing, humming or buzzing noises in the ears) and a feeling of fullness or pressure in the ear. It usually affects adults, and starts in middle age.

**How is Ménière's disease treated?**

Oral medications (tablets) are often used as the first treatment for Ménière's disease. Other treatment options are also available (for example, injections into the ear or surgery). People with Ménière's disease are often advised to modify their diet, for example by reducing their intake of salt or caffeine, as these are thought to worsen the symptoms of the disease.

**What did we want to find out?**

We wanted to find out:

‐ whether there was evidence that any lifestyle or dietary modifications work at reducing the symptoms of Ménière's disease;

‐ whether the treatments might cause any harm.

**What did we do?**

We searched for studies that compared different types of lifestyle or dietary modifications to either no modifications or sham (placebo) treatment.

**What did we find?**

We found two studies, which included a total of 274 people. One lasted for three months and the other lasted for two years. They assessed different types of lifestyle and dietary changes.

One study looked at the use of 'specially processed cereals'. These are cereals that stimulate the body to produce antisecretory factor ‐ a protein that acts to reduce fluid production and decrease inflammation. Vertigo was not specifically assessed in this study, so we do not know if the cereals made any difference to this symptom. The study authors did not report whether there were any harms associated with the cereals.

One study looked at whether sleeping in a darkened room or drinking plenty of water affected the symptoms of Ménière's disease. It was unclear whether these changes made a difference to vertigo symptoms, and the study did not report on potential harms of these interventions.

**What are the limitations of the evidence?**

We have very little confidence in the evidence because the studies conducted were small and had problems in their conduct, which means that the results may be unreliable. We found very little information on how the symptoms of Ménière's disease were affected by the different changes, and no information on potential harms. We also did not find any studies that considered other, more commonly used dietary changes ‐ such as reducing the intake of salt or caffeine. Large, well‐conducted studies are needed to try and work out whether different lifestyle and dietary modifications are effective.

**How up‐to‐date is this evidence?**

This evidence is up‐to‐date to September 2022.

## Summary of findings

**Summary of findings 1:** Abundant water intake compared to no treatment for Ménière’s disease

**Abundant water intake compared to no treatment for Ménière’s disease**						
**Patient or population:** Ménière’s disease **Setting:** outpatients **Intervention:** abundant water intake **Comparison:** no treatment						
**Outcomes**	Anticipated absolute effects^*^ (95% CI)		**Relative effect (95% CI)**	**№ of participants (studies)**	**Certainty of the evidence (GRADE)**	**Comments**
	**Risk with no treatment/placebo**	**Risk with abundant water intake**				
Improvement in frequency of vertigo Assessed with: number of participants with no vertigo attacks from 18 to 24 months	Study population		RR 1.50 (1.18 to 1.91)	140 (1 RCT)	⊕⊝⊝⊝ **very low**^1^	Abundant water intake may result in an increase in the proportion of people who report no vertigo episodes at > 12 months, but the evidence is very uncertain.
	543 per 1000	814 per 1000 (641 to 1000)				
Change in vertigo	No study assessed this outcome					
Serious adverse events	No study assessed this outcome					
*The risk in the intervention group (and its 95% confidence interval) is based on the assumed risk in the comparison group and the **relative effect** of the intervention (and its 95% CI). **CI:** confidence interval; **RCT:** randomised controlled trial; **RR:** risk ratio						
**GRADE Working Group grades of evidence** **High certainty:** we are very confident that the true effect lies close to that of the estimate of the effect. **Moderate certainty:** we are moderately confident in the effect estimate: the true effect is likely to be close to the estimate of the effect, but there is a possibility that it is substantially different. **Low certainty:** our confidence in the effect estimate is limited: the true effect may be substantially different from the estimate of the effect. **Very low certainty:** we have very little confidence in the effect estimate: the true effect is likely to be substantially different from the estimate of effect.						

^1^Unblinded study. Potential for selective reporting for this outcome. Outcome considers complete resolution of vertigo only, not 'any improvement'. Optimal information size was not reached, taken as > 300 events for dichotomous outcomes as a rule of thumb.

**Summary of findings 2:** Sleeping in darkness compared to no treatment for Ménière’s disease

**Sleeping in darkness compared to no treatment for Ménière’s disease**						
**Patient or population:** Ménière’s disease **Setting:** outpatients **Intervention:** sleeping in darkness **Comparison:** no treatment						
**Outcomes**	Anticipated absolute effects^*^ (95% CI)		**Relative effect (95% CI)**	**№ of participants (studies)**	**Certainty of the evidence (GRADE)**	**Comments**
	**Risk with no treatment/placebo**	**Risk with sleeping in darkness**				
Improvement in frequency of vertigo Assessed with: number of participants reporting no vertigo episodes between 18 and 24 months	Study population		RR 1.47 (1.15 to 1.89)	130 (1 RCT)	⊕⊝⊝⊝ Very low ^1^	Sleeping in darkness may result in an increase in the proportion of people who report no vertigo episodes at > 12 months, but the evidence is very uncertain.
	543 per 1000	798 per 1000 (624 to 1000)				
Change in vertigo	No study assessed this outcome					
Serious adverse events	No study assessed this outcome					
*The risk in the intervention group (and its 95% confidence interval) is based on the assumed risk in the comparison group and the **relative effect** of the intervention (and its 95% CI). **CI:** confidence interval; **RCT:** randomised controlled trial; **RR:** risk ratio						
**GRADE Working Group grades of evidence** **High certainty:** we are very confident that the true effect lies close to that of the estimate of the effect. **Moderate certainty:** we are moderately confident in the effect estimate: the true effect is likely to be close to the estimate of the effect, but there is a possibility that it is substantially different. **Low certainty:** our confidence in the effect estimate is limited: the true effect may be substantially different from the estimate of the effect. **Very low certainty:** we have very little confidence in the effect estimate: the true effect is likely to be substantially different from the estimate of effect.						

^1^Unblinded study. Potential for selective reporting for this outcome. Outcome considers complete resolution of vertigo only, not 'any improvement'. Optimal information size was not reached, taken as > 300 events for dichotomous outcomes as a rule of thumb.

**Summary of findings 3:** Specially processed cereals compared to placebo for Ménière’s disease

**Specially processed cereals compared to placebo for Ménière’s disease**						
**Patient or population:** Ménière’s disease **Setting:** outpatients **Intervention:** specially processed cereals **Comparison:** placebo						
**Outcomes**	Anticipated absolute effects^*^ (95% CI)		**Relative effect (95% CI)**	**№ of participants (studies)**	**Certainty of the evidence (GRADE)**	**Comments**
	**Risk with no treatment/placebo**	**Risk with specially processed cereals**				
Improvement in vertigo	No study assessed this outcome					
Change in vertigo	No study assessed this outcome					
Serious adverse events	No study assessed this outcome					
*The risk in the intervention group (and its 95% confidence interval) is based on the assumed risk in the comparison group and the **relative effect** of the intervention (and its 95% CI). **CI:** confidence interval						
**GRADE Working Group grades of evidence** **High certainty:** we are very confident that the true effect lies close to that of the estimate of the effect. **Moderate certainty:** we are moderately confident in the effect estimate: the true effect is likely to be close to the estimate of the effect, but there is a possibility that it is substantially different. **Low certainty:** our confidence in the effect estimate is limited: the true effect may be substantially different from the estimate of the effect. **Very low certainty:** we have very little confidence in the effect estimate: the true effect is likely to be substantially different from the estimate of effect.						

## Background

### Description of the condition

Ménière's disease was first described by Prosper Ménière in 1861 as a condition characterised by episodes of vertigo, associated with hearing loss and tinnitus (Baloh 2001). Sufferers may also report a feeling of fullness in the affected ear. Typically, it initially affects one ear, although some individuals may progress to develop bilateral disease. A hallmark of the condition is that symptoms are intermittent ‐ occurring as discrete attacks that last from minutes to several hours, then resolve. However, over time there is usually a gradual deterioration in hearing, and there may be progressive loss of balance function, leading to chronic dizziness.

The diagnosis of Ménière's disease is challenging, due to the episodic nature of the condition, clinical heterogeneity and the lack of a 'gold standard' diagnostic test. Even the agreed, international classification system has scope for two categories of diagnosis – 'definite' and 'probable' (Lopez‐Escamez 2015). In brief, a diagnosis of definite Ménière's disease requires at least two episodes of vertigo, each lasting 20 minutes to 12 hours, together with audiometrically confirmed hearing loss and fluctuating aural symptoms (reduction in hearing, tinnitus or fullness) in the affected ear. 'Probable' Ménière's disease includes similar features, but without the requirement for audiometry to diagnose hearing loss, and with scope for the vertigo episodes to last longer (up to 24 hours). Both categories ('definite' and 'probable') require that the symptoms are not more likely to be due to an alternative diagnosis, due to the recognised challenges in distinguishing between balance disorders. 

Given the difficulties in diagnosis, the true incidence and prevalence of the disease are difficult to ascertain. A population‐based study in the UK using general practice data estimated the incidence to be 13.1 per 100,000 person‐years (Bruderer 2017), and the prevalence of the disease has been estimated at 190 per 100,000 people in the US (Harris 2010). It is a disorder of mid‐life, with diagnosis typically occurring between the ages of 30 and 60 (Harcourt 2014). Some studies report a slight female preponderance, and there may be a familial association, with approximately 10% of patients reporting the presence of the disease in a first, second or third degree relative (Requena 2014).

The underlying cause of Ménière's disease is usually unknown. Ménière's disease has been associated with an increase in the volume of fluid in the inner ear (endolymphatic hydrops). This may be caused by the abnormal production or resorption of endolymph (Hallpike 1938; Yamakawa 1938). However, it is not clear whether this is the underlying cause of the condition, or merely associated with the disease. Some authors have proposed other underlying causes for Ménière's disease, including viral infections (Gacek 2009), allergic (Banks 2012) or autoimmune disease processes (Greco 2012). A genetic predisposition has also been noted (Chiarella 2015). Occasionally, the symptoms may be secondary to a known cause (such as a head injury or other inner ear disorder) – in these cases it may be referred to as Ménière's syndrome.

Although Ménière's disease is relatively uncommon, it has a profound impact on quality of life. The unpredictable, episodic nature of the condition and severe, disabling attacks of vertigo cause a huge amount of distress. Quality of life (including physical and psychosocial aspects) is significantly reduced for those with Ménière's disease (Söderman 2002). The costs of the condition are also considerable, both in relation to medical interventions (appointments, diagnostic tests and treatments) and loss of productivity or sick days for those affected by the condition (Tyrrell 2016).

### Description of the intervention

A variety of different interventions have been proposed to treat people with Ménière's disease. These include dietary or lifestyle changes, oral treatments, treatments administered by injection into the ear (intratympanic) and surgical treatments. This review focuses on lifestyle and dietary modifications. 

Lifestyle interventions have been proposed to be of benefit in Ménière's disease. Lifestyle medicine has been defined as the "evidence‐based practice of assisting individuals and families to adopt and sustain behaviors that can improve health and quality of life" (Lianov 2010). Therefore for the purposes of this review we consider lifestyle interventions to include any intervention that aims to modify physical activity, sleep patterns or stress management. This may include psychological interventions, such as stress counselling or cognitive behavioural therapy. Some of these interventions may require input from a therapist, whilst others can be self‐delivered.

Many dietary changes have been suggested to benefit patients with Ménière's disease. Salt restriction has been suggested to be of benefit for many years, with dietary intake of sodium usually recommended to be less than 2000 mg per day (Sharon 2015). A survey of UK‐based ENT surgeons found that restriction of salt was the second most common 'medical intervention' recommended to patients with Ménière's disease, after betahistine (Smith 2005). Restriction of caffeine and alcohol has also been said to benefit individuals with Ménière's disease, although there does not appear to be a consensus on the level of intake that is acceptable. 

More recently, intake of specially processed cereals has been suggested as a potential therapy for Ménière's disease. These are eaten as a dietary supplement, and have also been used in the treatment of inflammatory bowel disease (Bjorck 2000). Other dietary changes have been proposed, such as following a gluten‐free diet (di Berardino 2012). 

At present, there is no agreement on which is the ideal treatment for people with Ménière's disease – consequently there is no 'gold standard' treatment with which to compare these interventions. 

### How the intervention might work

As the underlying cause of Ménière's disease is poorly understood, so too are the ways in which the interventions may work.

Restriction of salt, caffeine or alcohol may work by changing fluid balance, thereby affecting the volume of endolymphatic fluid. There has also been interest in 'specially processed cereals', which are thought to promote the release of a protein known as antisecretory factor. This protein was initially identified in the intestine (and the pituitary gland) during diarrhoeal diseases, where it acts to reduce excessive fluid secretion. However, it is now thought to affect water and electrolyte balance more widely (Lange 2001; Ulgheri 2010). 

Psychological factors have been recognised to play a part in Ménière's disease (van Cruijsen 2003), and many patients identify stress as a trigger for their attacks (Kirby 2012). Stress management, improving sleep patterns and counselling may help patients to manage anxiety or mood disturbance associated with their disease and therefore assist with improving overall quality of life. They may also help patients to develop coping strategies for their symptoms, including reducing the distress associated with acute vertigo and the communication difficulties associated with hearing loss. 

### Why it is important to do this review

Balance disorders can be difficult to diagnose and treat. There are few specific diagnostic tests, a variety of related disorders with similar symptoms and a limited number of interventions that are known to be effective. To determine which topics within this area should be addressed with new or updated systematic reviews we conducted a scoping and prioritisation process, involving stakeholders (https://ent.cochrane.org/balance-disorders-ent). Ménière's disease was ranked as one of the highest priority topics during this process (along with vestibular migraine and persistent postural perceptual dizziness). 

Although Ménière's disease is a relatively uncommon condition, the significant impact it has on quality of life demonstrates the clear importance of identifying effective interventions to alleviate the symptoms. There is considerable variation in the management of Ménière's disease on both a national and international scale, with a lack of consensus about appropriate first‐line and subsequent therapies. 

This review is part of a suite of six that consider different interventions for Ménière's disease. Through these reviews, we hope to provide a thorough summary of the efficacy (benefits and harms) of the different treatment options, to support people with Ménière's disease (and healthcare professionals) when making decisions about their care. 

## Objectives

To evaluate the benefits and harms of lifestyle and dietary interventions versus placebo or no treatment in people with Ménière's disease.

## Methods

### Criteria for considering studies for this review

#### Types of studies

We included randomised controlled trials (RCTs) and quasi‐randomised trials (where trials were designed as RCTs, but the sequence generation for allocation of treatment used methods such as alternate allocation, birth dates etc). 

Ménière's disease is known to fluctuate over time, which may mean that cross‐over trials are not an appropriate study design for this condition. However, no cross‐over RCTs or cluster‐RCTs were identified as relevant for inclusion in this review.

We included studies reported as full‐text, those published as conference abstracts only and unpublished data. 

Ménière's disease is characterised by episodic balance disturbance ‐ the frequency of attacks may change over time (Huppert 2010). For studies to obtain accurate estimates of the effect of different interventions, we considered that follow‐up of participants should be for at least three months ‐ to ensure that participants are likely to have experienced a number of attacks during the follow‐up period. Studies that followed up participants for less than three months were excluded from the review.

#### Types of participants

We included studies that recruited adult participants (aged 18 years or older) with a diagnosis of definite or probable Ménière's disease, according to the agreed criteria of the American Academy Otolaryngology ‐ Head and Neck Surgery (AAO‐HNS), the Japan Society for Equilibrium Research, the European Academy of Otology and Neurotology and the Bárány Society. These criteria are outlined in Appendix 1 and described in Lopez‐Escamez 2015. If studies used different criteria to diagnose Ménière's disease, we included them if those criteria were clearly analogous to those described in Lopez‐Escamez 2015. For example, studies that used earlier definitions of Ménière's disease (from the AAO‐HNS guidelines of 1995) were also included. 

We anticipated that most studies would include participants with active Ménière's disease. We did not exclude studies if the frequency of attacks at baseline was not reported or was unclear, but we planned to highlight if there were differences between studies that may impact on our ability to pool the data, or affect the applicability of our findings.

We excluded studies where participants had previously undergone destructive/ablative treatment for Ménière's disease in the affected ear (such as vestibular neurectomy, chemical or surgical labyrinthectomy), as we considered that they were unlikely to respond to interventions in the same way as those who had not undergone such treatment.

#### Types of interventions

We included the following interventions:

- Therapist‐delivered lifestyle interventions (including any lifestyle intervention that requires interaction with/guidance from a therapist).
- Self‐delivered lifestyle interventions (including any lifestyle intervention that can be self‐delivered, such as through reading a booklet, watching a video etc.).
- Modification of salt intake.
- Modification of caffeine intake.
- Modification of alcohol intake.
- Modification of water intake.
- Dietary modifications (e.g. use of specially processed cereals, gluten free diet etc.).

The main comparisons were as follows:

- Therapist‐delivered lifestyle intervention versus placebo/no treatment.
- Self‐delivered lifestyle intervention versus placebo/no treatment.
- Reduction of salt intake versus placebo/no treatment.
- Reduction of caffeine intake versus placebo/no treatment.
- Reduction of alcohol intake versus placebo/no treatment.
- Increase in water intake versus placebo/no treatment.
- Dietary modifications versus placebo/no treatment.

##### Concurrent treatments

There were no limits on the type of concurrent treatments used, providing these were used equally in each arm of the study. We planned to pool studies that included concurrent treatments with those where participants did not receive concurrent treatment. We planned to conduct subgroup analysis to determine whether the effect estimates may be different in those receiving additional treatment. However, due to the small number of studies included in the review this was not possible (see Subgroup analysis and investigation of heterogeneity). 

#### Types of outcome measures

We assessed outcomes at the following time points: 

- 3 to < 6 months;
- 6 to ≤ 12 months;
- > 12 months.

The exception was for adverse event data, when we used the longest time period of follow‐up. 

We searched the COMET database for existing core outcome sets of relevance to Ménière's disease and vertigo, but were unable to find any published core outcome sets. We therefore conducted a survey of individuals with experience of (or an interest in) balance disorders to help identify the outcomes that should be prioritised. The review author team used the results of this survey to inform the choice of outcome measures in this review. 

We analysed the following outcomes in the review, but did not use them as a basis for including or excluding studies.

##### Primary outcomes

- Improvement in vertigo
  - Measured as a dichotomous outcome (improved/not improved), according to self‐report, or according to a change of a specified score (as described by the study authors) on a vertigo rating scale.
- Change in vertigo
  - Measured as a continuous outcome, to identify the extent of change in vertigo symptoms.
- Serious adverse events
  - Including any event that causes death, is life‐threatening, requires hospitalisation, results in disability or permanent damage, or in congenital abnormality. Measured as the number of participants who experience at least one serious adverse event during the follow‐up period.

Vertigo symptoms comprise a variety of different features, including frequency of episodes, duration of episodes and severity/intensity of the episodes. Where possible, we included data for the vertigo outcomes that encompassed all of these three aspects (frequency, duration and severity/intensity of symptoms). However, we anticipated that these data may not be available from all studies. We therefore extracted data on the frequency of vertigo episodes as an alternative measure for these outcomes. 

##### Secondary outcomes

- Disease‐specific health‐related quality of life
  - Measured with the Dizziness Handicap Inventory (DHI, Jacobsen 1990), a validated measurement scale in widespread use. If data from the DHI were unavailable we extracted data from alternative validated measurement scales, according to the order of preference described in the list below (based on the validity of the scales for this outcome):
    - DHI short form (Tesio 1999);
    - DHI screening tool (Jacobsen 1998);
    - Vertigo Handicap Questionnaire (Yardley 1992a);
    - Meniere's Disease Patient Oriented Symptoms Inventory (MD POSI, Murphy 1999);
    - University of California Los Angeles Dizziness Questionnaire (UCLADQ, Honrubia 1996);
    - AAO‐HNS Functional Level Scale (FLS, AAO‐HNS 1995).
- Hearing
  - Measured with pure tone audiometry and reported as the change in pure tone average (PTA), or (alternatively) by patient report, if data from PTA were not available.
- Tinnitus
  - Measured using any validated, patient‐reported questionnaire relating to the impact of tinnitus, for example the Tinnitus Handicap Inventory (THI, Newman 1996) or the Tinnitus Functional Index (TFI, Meikle 2012).
- Other adverse effects
  - We reported the number of participants who discontinued the intervention due to adverse effects, or for other reasons.
  - We also used an exploratory approach to adverse events, and recorded any specific adverse events described in the studies.

### Search methods for identification of studies

The Cochrane ENT Information Specialist conducted systematic searches for randomised controlled trials and controlled clinical trials in October 2021 and September 2022. There were no language, publication year or publication status restrictions. The date of the latest search was 14 September 2022.

#### Electronic searches

The Information Specialist searched:

- the Cochrane ENT Trials Register (search via the Cochrane Register of Studies to 14 September 2022);
- the Cochrane Central Register of Controlled Trials (CENTRAL) (search via the Cochrane Register of Studies to 14 September 2022);
- Ovid MEDLINE(R) Epub Ahead of Print, In‐Process & Other Non‐Indexed Citations, Ovid MEDLINE(R) Daily and Ovid MEDLINE(R) (1946 to 14 September 2022);
- Ovid Embase (1974 to 14 September 2022);
- Web of Knowledge, Web of Science (1945 to 14 September 2022);
- ClinicalTrials.gov, www.clinicaltrials.gov (to 14 September 2022);
- World Health Organization (WHO) International Clinical Trials Registry Platform (ICTRP), https://trialsearch.who.int/ (to 14 September 2022).

The Information Specialist modelled subject strategies for databases on the search strategy designed for CENTRAL. The strategy has been designed to identify all relevant studies for a suite of reviews on various interventions for Ménière's disease. Where appropriate, they were combined with subject strategy adaptations of the highly sensitive search strategy designed by Cochrane for identifying randomised controlled trials and controlled clinical trials (as described in the *Cochrane Handbook for Systematic Reviews of Interventions* Version 5.1.0, Box 6.4.b, Handbook 2011). Search strategies for major databases including CENTRAL are provided in Appendix 2.

#### Searching other resources

We scanned the reference lists of identified publications for additional trials and contacted trial authors where necessary. In addition, the Information Specialist searched Ovid MEDLINE to retrieve existing systematic reviews relevant to this systematic review, so that we could scan their reference lists for additional trials. In addition, the Information Specialist ran a non‐systematic search of Google Scholar to identify trials not published in mainstream journals.

We did not perform a separate search for adverse effects. We considered adverse effects described in included studies only.

### Data collection and analysis

#### Selection of studies

The Cochrane ENT Information Specialist used the first two components of Cochrane's Screen4Me workflow to help assess the search results: 

1. Known assessments – a service that matches records in the search results to records that have already been screened in Cochrane Crowd and been labelled as 'a RCT' or as 'not a RCT'. 
2. The machine learning classifier (RCT model) (Wallace 2017), available in the Cochrane Register of Studies (CRS‐Web), which assigns a probability of being a true RCT (from 0 to 100) to each citation. Citations that were assigned a probability score below the cut‐point at a recall of 99% were assumed to be non‐RCTs. We manually dual screened the results for those that scored on or above the cut‐point. 

At least two review authors (BG, KG, AL, KW) independently screened the remaining titles and abstracts using Covidence, to identify studies that may be relevant for the review. Any discrepancies were resolved by consensus, or by retrieving the full text of the study for further assessment. 

We obtained the full text for any study that was considered possibly relevant and two authors (BG, KG, AL, KW) again independently checked this to determine whether it met the inclusion criteria for the review. Any differences were resolved by discussion and consensus, or through recourse to a third author if necessary. 

We excluded any studies that were retrieved in full text but subsequently deemed to be inappropriate for the review (according to the inclusion/exclusion criteria), according to the main reason for exclusion. 

The unit of interest for the review is the study, therefore multiple papers or reports of a single study are grouped together under a single reference identification. The process for study selection is recorded in Figure 1. 

**1:**
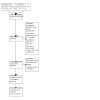
Flow chart of study retrieval and selection.

##### Screening eligible studies for trustworthiness

We assessed studies meeting our inclusion criteria for trustworthiness using a screening tool developed by Cochrane Pregnancy and Childbirth. This tool includes specified criteria to identify studies that are considered sufficiently trustworthy to be included in the review (see Appendix 3 and Figure 2). If studies were assessed as being potentially 'high‐risk', we attempted to contact the study authors to obtain further information or address any concerns. We planned to exclude studies from the main analyses of the review if there were persisting concerns over trustworthiness, or we were unable to contact the authors. 

**2:**
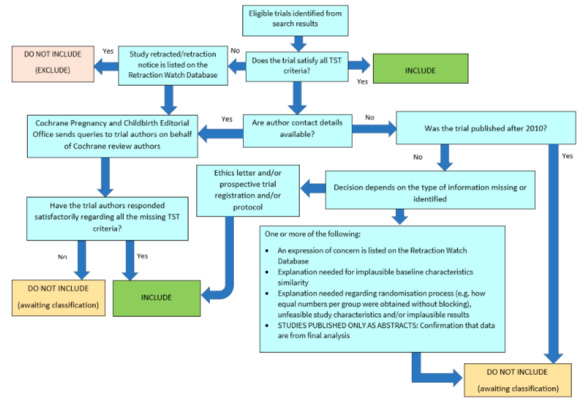
The Cochrane Pregnancy and Childbirth Trustworthiness Screening Tool

However, both of the included studies had some concerns when using the tool ‐ due to the large effect size seen for the primary outcome measures, despite the relatively small size of the trials, and also because only limited baseline information was reported (preventing full assessment of the randomisation process). 

We had not anticipated this issue when drafting the protocol for our review, but it is likely to be a widespread issue for reviews that incorporate older studies, and has been a persistent problem through this suite of reviews on Ménière's disease.

There are several possible explanations for the large number of studies across the suite that had concerns when using the tool. One is that there are issues with the trustworthiness of the studies identified in this review, and the data included may not give reliable estimates of the true effect. Alternatively, the trustworthiness screening tool may be excessively sensitive, and flag studies that are trustworthy, but where information has not been fully reported. We note that this tool (and others used for the same purpose) has not yet been validated for use. 

We therefore took the decision to include the studies in the review, despite the potential concerns over trustworthiness. The uncertainty in the results is captured as part of our GRADE rating in the certainty of the evidence, using the domain 'study limitations'. 

#### Data extraction and management

Two review authors (BG, AL) independently extracted outcome data from each study using a standardised data collection form. Where a study had more than one publication, we retrieved all publications to ensure that we had a complete data set. We checked any discrepancies in the data extracted by the two authors against the original reports, and resolved differences through discussion and consensus. If required, we contacted the study authors for clarification.

We extracted data on the key characteristics of the studies, including the following information:

- study design, duration of the study, number of study centres and location, study setting and dates of the study;
- information on the participants, including the number randomised, those lost to follow‐up or withdrawn, the number analysed, the age of participants, gender, severity of the condition, diagnostic criteria used, inclusion and exclusion criteria for the individual studies;
- details of the intervention, comparator, and concomitant treatments or excluded medications;
- the outcomes specified and reported by the study authors, including the time points;
- funding for the study and any conflicts of interest for the study authors;
- information required to assess the risk of bias in the study, and to enable GRADE assessment of the evidence.

Once the extracted data were checked and any discrepancies resolved, a single author (KW) transferred the information to Review Manager 5 (RevMan 2020).

The primary effect of interest for this review is the effect of treatment assignment (which reflects the outcomes of treatment for people who were assigned to the intervention) rather than a per protocol analysis (the outcomes of treatment only for those who completed the full course of treatment as planned). For the outcomes of interest in this review, we extracted the findings from the studies on an available case basis, i.e. all available data from all participants at each time point, based on the treatment to which they were randomised. This was irrespective of compliance, or whether participants had received the intervention as planned.

In addition to extracting pre‐specified information about study characteristics and aspects of methodology relevant to risk of bias, we extracted the following summary statistics for each study and outcome:

- For continuous data: the mean values, standard deviation and number of patients for each treatment group at the different time points for outcome measurement. Where change‐from‐baseline data were not available, we extracted the values for endpoint data instead. If values for the individual treatment groups were not reported, where possible we extracted summary statistics (e.g. mean difference) from the studies.
- For binary data: we extracted information on the number of participants experiencing an event, and the number of participants assessed at that time point. If values for the individual treatment groups were not reported, where possible we extracted summary statistics (e.g. risk ratio) from the studies.
- For ordinal scale data: if the data appeared to be normally distributed, or if the analysis performed by the investigators indicated that parametric tests are appropriate, then we treated the outcome measure as continuous data. Alternatively, if data were available, we converted these to binary data for analysis ‐ for example, for analysis of improvement in disease‐specific health‐related quality of life, when rated using the AAO‐HNS 1995 Functional Level Scale.
- For time‐to‐event data: we did not identify any time‐to‐event data for the outcomes specified in the review. 

If necessary, we converted data found in the studies to a format appropriate for meta‐analysis, according to the methods described in the *Cochrane Handbook for Systematic Reviews of Interventions* (Handbook 2021). 

We pre‐specified time points of interest for the outcomes in this review. Where studies reported data at multiple time points, we planned to take the longest available follow‐up point within each of the specific time frames. However, the studies included in this review only reported at one time‐point.

#### Assessment of risk of bias in included studies

Two authors (BG, AL) undertook assessment of the risk of bias of the included studies independently, with the following taken into consideration, as guided by the *Cochrane Handbook for Systematic Reviews of Interventions* (Handbook 2011):

- sequence generation;
- allocation concealment;
- blinding;
- incomplete outcome data;
- selective outcome reporting; and
- other sources of bias.

We used the Cochrane risk of bias tool (Handbook 2011), which involves describing each of these domains as reported in the study and then assigning a judgement about the adequacy of each entry: 'low', 'high' or 'unclear' risk of bias.

#### Measures of treatment effect

We summarised the effects of the majority of dichotomous outcomes (e.g. serious adverse effects) as risk ratios (RR) with 95% confidence intervals (CIs). We have also expressed the results as absolute numbers based on the pooled results and compared to the assumed risk in the summary of findings tables (Table 1; Table 2; Table 3) and full GRADE profiles (Table 4; Table 5; Table 6).

**1:** GRADE profile: Abundant water intake compared to no treatment for Ménière’s disease

**Certainty assessment**							**Number of participants**		**Effect**		**Certainty**	**Comment**
**№ of studies**	**Study design**	**Risk of bias**	**Inconsistency**	**Indirectness**	**Imprecision**	**Other considerations**	**Abundant water intake**	**No treatment**	**Relative** **(95% CI)**	**Absolute** **(95% CI)**		
**Improvement in frequency of vertigo (follow‐up: range > 12 months; assessed with: number of participants with no vertigo attacks from 18 to 24 months)**												
1	Randomised trials	Very serious^a,b^	Not serious	Serious^c^	Serious^d^	None	57/70 (81.4%)	38/70 (54.3%)	**RR 1.50** (1.18 to 1.91)	**271 more per 1000** (from 98 more to 494 more)	⨁◯◯◯ Very low	Abundant water intake may increase the proportion of people who experience complete resolution of vertigo, but the evidence is very uncertain.
**Change in hearing: improvement in hearing (follow‐up: range > 12 months; assessed with: number of participants with improvement of ≥ 10 dB on PTA)**												
1	Randomised trials	Serious^a^	Not serious	Not serious	Serious^d^	None	25/70 (35.7%)	5/70 (7.1%)	**RR 5.00** (2.03 to 12.31)	**286 more per 1000** (from 74 more to 808 more)	⨁⨁◯◯ Low	Abundant water intake may increase the number of people who experience an improvement in hearing.
**Other adverse events: discontinuation (follow‐up: range > 12 months; assessed with: number of participants excluded from follow‐up due to < 75% compliance)**												
1	Randomised trials	Serious^a^	Not serious	Serious^e^	Very serious^d,f^	None	5/70 (7.1%)	4/74 (5.4%)	**RR 1.32** (0.37 to 4.72)	**17 more per 1000** (from 34 fewer to 201 more)	⨁◯◯◯ Very low	There was little difference between the groups in the proportion of people who discontinued their allocated intervention, but the evidence is very uncertain.

**CI:** confidence interval; **RR:** risk ratio ^a^Unblinded study. ^b^Potential for selective reporting for this outcome. ^c^Outcome considers complete resolution of vertigo only, not 'any improvement'. ^d^Optimal information size was not reached, taken as > 300 events for dichotomous outcomes, as a rule of thumb. ^e^Only includes those with < 75% compliance. This may be greater than the number of participants who discontinue the intervention completely. ^f^Confidence interval ranges from potential benefit to potential harm from the intervention.

**2:** GRADE profile: Sleeping in darkness compared to no treatment

**Certainty assessment**							**№ of participants**		**Effect**		**Certainty**	**Comment**
**№ of studies**	**Study design**	**Risk of bias**	**Inconsistency**	**Indirectness**	**Imprecision**	**Other considerations**	**Sleeping in darkness**	**No treatment**	**Relative** **(95% CI)**	**Absolute** **(95% CI)**		
**Improvement in frequency of vertigo (follow‐up: range > 12 months; assessed with: number of participants reporting no vertigo between 18 and 24 months)**												
1	Randomised trials	Very serious^a,b^	Not serious	Serious^c^	Serious^d^	None	48/60 (80.0%)	38/70 (54.3%)	**RR 1.47** (1.15 to 1.89)	**255 more per 1000** (from 81 more to 483 more)	⨁◯◯◯ Very low	Sleeping in darkness may increase the proportion of people who experience complete resolution of vertigo, but the evidence is very uncertain.
**Change in hearing: improvement in hearing (follow‐up: range > 12 months; assessed with: number of participants with ≥ 10 dB improvement in hearing on PTA)**												
1	Randomised trials	Serious^a^	Not serious	Not serious	Serious^d^	None	19/60 (31.7%)	5/70 (7.1%)	**RR 4.43** (1.76 to 11.16)	**245 more per 1000** (from 54 more to 726 more)	⨁⨁◯◯ Low	Sleeping in darkness may increase the number of people who experience an improvement in hearing.
**Other adverse events: discontinuation (follow‐up: range > 12 months; assessed with: number of participants excluded from follow‐up due to < 75% compliance)**												
1	Randomised trials	Serious^a^	Not serious	Serious^e^	Serious^d^	None	14/74 (18.9%)	4/74 (5.4%)	**RR 3.50** (1.21 to 10.14)	**135 more per 1000** (from 11 more to 494 more)	⨁◯◯◯ Very low	There may be more chance that people allocated to sleeping in darkness will discontinue this intervention, but the evidence is very uncertain.

**CI:** confidence interval; **dB:** decibels; **PTA:** pure tone audiometry; **RR:** risk ratio ^a^Unblinded study. ^b^Potential for selective reporting for this outcome. ^c^Outcome considers complete resolution of vertigo only, not 'any improvement'. ^d^Optimal information size was not reached, taken as > 300 events for dichotomous outcomes or > 400 participants for continuous outcomes, as a rule of thumb. ^e^Only includes those with < 75% compliance. This may be greater than the number of participants who discontinue the intervention completely.

**3:** GRADE profile: Specially processed cereals compared to placebo

**Certainty assessment**							**№ of participants**		**Effect**		**Certainty**	**Comment**
**№ of studies**	**Study design**	**Risk of bias**	**Inconsistency**	**Indirectness**	**Imprecision**	**Other considerations**	**Specially processed cereals**	**Placebo**	**Relative** **(95% CI)**	**Absolute** **(95% CI)**		
**Change in disease‐specific health‐related quality of life (follow‐up: range 3 months to < 6 months; assessed with: AAO‐HNS Functional Level Scale; from: 1 to 6, higher scores = worse quality of life)**												
1	Randomised trials	Serious^a^	Not serious	Serious^b^	Serious^c^	None	27	24	‐	MD **1.35 points lower** (2.08 lower to 0.62 lower)	⨁◯◯◯ Very low	The evidence is very uncertain about the effect of specially processed cereals on disease‐specific quality of life.

**AAO‐HNS:** American Academy of Otolaryngology ‐ Head and Neck Surgery; **CI:** confidence interval; **MD:** mean difference ^a^Multiple domains at unclear risk of bias, leading to some concerns. ^b^It is unclear whether all participants met the AAO‐HNS 1995 criteria for a diagnosis of Ménière's disease. ^c^Optimal information size was not reached, taken as > 400 participants for continuous outcomes, as a rule of thumb.

For continuous outcomes, we expressed treatment effects as a mean difference (MD) with standard deviation (SD). We did not need to use the standardised mean difference to pool any data. 

#### Unit of analysis issues

Ménière's disease is unlikely to be a stable condition, and interventions may not have a temporary effect. If cross‐over trials are identified then we planned to use the data from the first phase of the study only. If cluster‐randomised trials were identified then we would have ensured that analysis methods were used to account for clustering in the data (Handbook 2021). However, we did not identify any cross‐over studies, or cluster‐randomised trials.

We did identify one study with four arms (Kitahara 2016). One arm was not relevant for this review, and is included in a separate review of surgical interventions for Ménière's disease (Webster 2021a). The remaining arms comprised two active interventions, and one comparator. As both active interventions were relevant for separate comparisons in this review (sleeping in darkness and abundant water intake), we have included the 'no treatment' arm as the comparator for both of the active interventions. 

#### Dealing with missing data

We planned to contact study authors via email whenever the outcome of interest was not reported, if the methods of the study suggest that the outcome had been measured. We did the same if not all data required were reported (for example, standard deviations), unless we were able to calculate them from other data reported by the study authors. 

#### Assessment of heterogeneity

We planned to assess clinical heterogeneity by examining the included studies for potential differences between them, as well as using the I^2^ statistic and the P value from the Chi^2^ test. However, each of the studies examined a different comparison, and we could not conduct any meta‐analysis.

#### Assessment of reporting biases

We assessed reporting bias as within‐study outcome reporting bias and between‐study publication bias.

##### Outcome reporting bias (within‐study reporting bias)

We assessed within‐study reporting bias by comparing the outcomes reported in the published report against the study protocol or trial registry, whenever this could be obtained. If the protocol or trial registry entry was not available, we compared the outcomes reported to those listed in the methods section. If results are mentioned but not reported adequately in a way that allows analysis (e.g. the report only mentions whether the results were statistically significant or not), bias in a meta‐analysis is likely to occur. We then sought further information from the study authors. If no further information was found, we noted this as being a 'high' risk of bias with the risk of bias tool. If there was insufficient information to judge the risk of bias we noted this as an 'unclear' risk of bias (Handbook 2011). 

##### Publication bias (between‐study reporting bias)

We did not have sufficient studies to create funnel plots for any analysis. Any studies identified through trial registries and other sources (Searching other resources) that remain unpublished are noted in the Ongoing studies section. 

#### Data synthesis

##### Meta‐analysis of numerical data

We planned to conduct a meta‐analysis of numerical data where possible and appropriate (if participants, interventions, comparisons and outcomes are sufficiently similar in the trials identified). However, we only identified a single study for each comparison in this review, therefore no meta‐analysis was possible. 

##### Synthesis using other methods

If we were unable to pool numerical data in a meta‐analysis for one or more outcomes we planned to provide a synthesis of the results using alternative methods, following the guidance in chapter 12 of the Handbook 2021. However, this was not necessary, as results were provided by a single study. 

#### Subgroup analysis and investigation of heterogeneity

If statistical heterogeneity was identified for any comparison, we planned to assess this considering the following subgroups:

- Different types of lifestyle management.
- Use of concomitant treatment.
- Diagnosis of Ménière's disease.

However, due to the paucity of data available, we did not carry out any subgroup analysis. 

#### Sensitivity analysis

We planned to carry out a number of sensitivity analyses for the primary outcomes in this review. However, the paucity of data and the lack of meta‐analyses has meant that this was not possible. 

We intended to carry out sensitivity analyses for the primary outcomes only, considering:

- the use of a fixed‐effect model instead of a random‐effects model;
- the diagnostic criteria used for Ménière's disease;
- the inclusion/exclusion of studies with identified concerns using the Cochrane Pregnancy and Childbirth Group Screening Tool.

#### Summary of findings and assessment of the certainty of the evidence

Two independent authors (KG, KW) used the GRADE approach to rate the overall certainty of evidence using GRADEpro GDT (https://gradepro.org/) and the guidance in chapter 14 of the *Cochrane Handbook for Systematic Reviews of Interventions* (Handbook 2021). Disagreements were resolved through discussion and consensus. The certainty of evidence reflects the extent to which we are confident that an estimate of effect is correct, and we have applied this in the interpretation of results. There are four possible ratings: high, moderate, low and very low. A rating of high certainty of evidence implies that we are confident in our estimate of effect and that further research is very unlikely to change our confidence in the estimate of effect. A rating of very low certainty implies that any estimate of effect obtained is very uncertain.

The GRADE approach rates evidence from RCTs that do not have serious limitations as high certainty. However, several factors can lead to the downgrading of the evidence to moderate, low or very low. The degree of downgrading is determined by the seriousness of these factors:

- Study limitations (risk of bias):
  - This was assessed using the rating from the Cochrane risk of bias tool for the study or studies included in the analysis. We rated down either one or two levels, depending on the number of domains that had been rated at high or unclear risk of bias. 
- Inconsistency:
  - This was assessed using the I^2^ statistic and the P value for heterogeneity for all meta‐analyses, as well as by visual inspection of the forest plot. For results based on a single study we rated this domain as no serious inconsistency.
- Indirectness of evidence:
  - We took into account whether there were concerns over the population included in these study or studies for each outcome, as well as whether additional treatments were offered that may impact on the efficacy of the intervention under consideration. 
- Imprecision:
  - We took into account the sample size and the width of the confidence interval for each outcome. If the sample size did not meet the optimal information size (i.e. < 400 people for continuous outcomes or < 300 events for dichotomous outcomes), or the confidence interval crossed the small effect threshold we rated down one level. If the sample size did not meet the optimal information size and the confidence interval includes both potential harm and potential benefit we rated down twice. We also rated down twice for very tiny studies (e.g. 10 to 15 participants in each arm), regardless of the estimated confidence interval.
- Publication bias:
  - We considered whether there were likely to be unpublished studies that may impact on our confidence in the results obtained. 

We used a minimally contextualised approach, and rated the certainty in the interventions having an important effect (Zeng 2021). Where possible, we used agreed minimally important differences (MIDs) for continuous outcomes as the threshold for an important difference. Where no MID was identified, we provide an assumed MID based on agreement between the authors. For dichotomous outcomes, we looked at the absolute effects when rating imprecision, but also took into consideration the GRADE default approach (rating down when a RR crosses 1.25 or 0.80). We have justified all decisions to downgrade the certainty of the evidence using footnotes, and added comments to aid the interpretation of the findings, where necessary. 

We planned to provide a summary of findings tables for the following comparisons:

- reduction of salt intake versus placebo/no treatment;
- reduction of caffeine intake versus placebo/no treatment;
- reduction of alcohol intake versus placebo/no treatment.

However, as we did not identify data for these comparisons then we have presented a summary of findings table for the other comparisons included in the review. We have included all primary outcomes in the summary of findings tables.

## Results

### Description of studies

#### Results of the search

The searches in September 2022 retrieved a total of 4434 records. This reduced to 3408 after the removal of duplicates. The Cochrane ENT Information Specialist sent all 3408 records to the Screen4Me workflow. The Screen4Me workflow identified 122 records as having previously been assessed: 83 had been rejected as not RCTs and 39 had been assessed as possible RCTs. The RCT classifier rejected an additional 1427 records as not RCTs (with 99% sensitivity). We did not send any records to the Cochrane Crowd for assessment. Following this process, the Screen4Me workflow had rejected 1510  records and identified 1898 possible RCTs for title and abstract screening. 

**Table:** 

** **	**Possible RCTs**	**Rejected**
Known assessments	39	83
RCT classifier	1859	1427
Total	1898	1510

We identified 89 additional duplicates. We screened the titles and abstracts of the remaining 1809 records. We discarded 1786 records and assessed 23 full‐text records. 

We excluded 18 records (linked to 16 studies) with reasons recorded in the review (see Excluded studies). We included two completed studies (three records) where results were available. Two additional records are listed as ongoing studies (see Characteristics of ongoing studies for details).

#### Included studies

We included two RCTs (Hanner 2010; Kitahara 2016). Details of the individual studies can be found in the Characteristics of included studies.

##### Study design

Both included studies were parallel‐group randomised controlled trials. The duration of follow‐up ranged from three months (Hanner 2010) to two years (Kitahara 2016). One trial was conducted in Japan (Kitahara 2016) and the other in Sweden (Hanner 2010).

##### Participants

Kitahara 2016 recruited adult participants with a diagnosis of definite Ménière's disease, according to the AAO‐HNS 1995 criteria. Hanner 2010 stated that participants with "definite Ménière's disease" were included, and cites the AAO‐HNS 1995 guidelines elsewhere in the article. However, there is no explicit description of the use of the AAO‐HNS 1995 criteria for inclusion in the study. Although the majority of participants in the Hanner 2010 study were adults, we note that the inclusion criteria were from age 12 to 80, and it appears that at least one 12‐year‐old was included in the study. We therefore have some uncertainty regarding the population included in this study. 

###### Features of Ménière's disease

The study Kitahara 2016 included individuals with unilateral Ménière's disease, in whom three to six months of medical treatment had not improved their symptoms. The treatment used before entry to the study varied for each individual, but included diuretics, betahistine, diphenidol, dimenhydrinate and diazepam. The mean duration of Ménière's disease in the participants was approximately 2.5 years, and the average attack frequency at baseline was 1.6 to 1.7 attacks per month. 

Hanner 2010 included participants with a longer duration of Ménière's disease on average (12 to 14 years, but with a range from 1 to 37 years). No information was provided on the attack frequency at baseline or therapy used before entry to the trial. 

###### Background interventions

Participants in Kitahara 2016 all continued to receive "traditional oral medications" throughout the course of the trial. As described previously, this varied for individual participants, but may have included diuretics, betahistine, diphenidol, dimenhydrinate and/or diazepam. 

Hanner 2010 stated that "no changes were made in the patient's ordinary medication", but no information was provided regarding the types of medication used.

##### Interventions and comparisons

###### Abundant water intake versus no treatment

Kitahara 2016 assessed this comparison, comparing water intake of 35 mL/kg/day plus standard, oral medications to oral medications alone. 

###### Sleeping in darkness versus no treatment

This comparison was also assessed by Kitahara 2016, comparing sleeping in darkness (defined as lying in bed in an unlit room less than 0.1 lux for six to seven hours per night) plus standard, oral medications to oral medications alone. 

###### Specially processed cereals versus placebo

Hanner 2010 considered this comparison, and randomised participants to receive either specially processed cereals ‐ which are intended to promote an increase in production of anti‐secretory factor ‐ or standard cereals, without anti‐secretory factor promoting activity. 

##### Outcomes

###### 1. Improvement in vertigo

For this outcome we included dichotomous data ‐ assessed as the proportion of participants whose vertigo had 'improved' or 'not improved'. 

####### 1.1. Global score

Neither study reported the improvement of vertigo using a global score that considered the frequency, duration and intensity of vertigo attacks. 

####### 1.2. Frequency

One study assessed improvement in vertigo frequency (Kitahara 2016), but actually considered complete resolution of vertigo, rather than any improvement. 

###### 2. Change in vertigo

This outcome included data on the change in vertigo using a continuous numerical scale. However, neither study reported on this outcome. 

###### 3. Serious adverse events

Neither study provided any details on how or whether serious adverse events were monitored and reported. 

###### 4. Disease‐specific health‐related quality of life

One study considered this outcome, and reported the Functional Level Scale (FLS) from the AAO‐HNS 1995 guidelines (Hanner 2010). This ranges from 1 to 6, with higher scores representing worse quality of life. 

###### 5. Hearing

Kitahara 2016 assessed hearing using pure tone audiometry at an average of four frequencies (0.5 kHz, 1 kHz, 2 kHz and 4 kHz). This was reported as a dichotomous outcome (improved/not improved) with a threshold of a decrease of 10 dB HL representing improvement. 

Hanner 2010 stated that audiometry was also used, but no numerical results are reported. 

###### 6. Tinnitus 

This outcome was not reported by any of the included studies. 

###### 7. Other adverse effects

None of the studies provided any details on how or whether adverse effects were monitored. No adverse events are reported in the studies. The study Kitahara 2016 provided some data regarding discontinuation of the intervention. The authors stated the following: "At their monthly check‐ups, patients were excluded if, according to a self‐declaration visual analogue scale, they had been <75% compliant with the rules for their group". We presume that this refers to following the instructions according to the study protocol. However, we note that some participants were also excluded in the control group, and it is not clear what would have constituted a lack of adherence in this group. 

#### Excluded studies

After assessing the full text, we excluded 16 articles (linked to 18 records) from this review. The main reason for exclusion for each article is listed below.

One study was a narrative review article on Ménière's disease (Richards 1971) and another was a systematic review that contained no primary data (Hussain 2018). One study was not a randomised controlled trial (NCT05355610).

Three studies assessed an incorrect population, and included participants who had conditions other than Ménière's disease (Faag 2017; Lee 2019; Shin 2017). One further study aimed to include participants with Ménière's disease, but required patients to self‐identify as having the disease in order to participate in the trial (Yardley 2006). No diagnostic criteria were applied at trial entry. We considered that this may result in a mixed population of participants, and therefore it could not be included in this review. 

Three studies assessed a form of traditional Chinese medicine (Hong 2011; Sun 2014; Zhong 2008), one considered transauricular cutaneous nerve stimulation (NCT05328895), and one considered vestibular rehabilitation (Cui 2022), which were not relevant interventions for this review. Two further studies did not follow up participants for at least three months (Garcia 2013; Liu 2020).

Finally, we identified two cross‐over trials (Ingvardsen 2016; Leong 2013). Although the study population and intervention (specially processed cereals) were relevant for the review, we were unable to extract data from the first period of the trial. In addition, one of the studies had an insufficient duration of follow‐up (two months; Ingvardsen 2016)

### Risk of bias in included studies

See Figure 3 for the risk of bias summary (our judgements about each risk of bias item for each included study) and Figure 4 for the risk of bias summary (our judgements about each risk of bias item presented as percentages across all included studies). Both studies had some concerns regarding the risk of bias, with at least one domain rated at high risk of bias.

**3:**
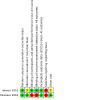
Risk of bias summary (our judgements about each risk of bias item for each included study).

**4:**
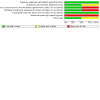
Risk of bias summary (our judgements about each risk of bias item presented as percentages across all included studies).

#### Allocation

Both included studies reported an adequate method to generate a random sequence (either simple drawing of lots, or computer‐generated random numbers). However, only Kitahara 2016 described the methods used to ensure concealment of group allocation, with Hanner 2010 being rated at unclear risk for this domain. 

#### Blinding

Kitahara 2016 was an open‐label study, with no attempts made to blind participants to the intervention received. This may be understandable, given the nature of the interventions (sleeping in darkness and abundant water intake), although it still presents a risk of performance bias. Study personnel and outcome assessors were also not blinded to the group allocation for each participant. We therefore rated it at high risk of performance and detection bias. 

Hanner 2010 used a placebo intervention to blind participants to their group allocation, but it was unclear whether study personnel were also blinded, therefore we rated the risk of performance bias as unclear. As the primary outcome was assessed by the participants themselves we considered this study to be at low risk of detection bias. 

#### Incomplete outcome data

We rated both studies at low risk of attrition bias.

#### Selective reporting

We rated both studies at high risk of selective reporting bias. We did identify a prospective trial registration for the study Kitahara 2016. However, the only outcome listed here was 'all cause mortality'. Therefore we could not fully assess whether the study was reported according to a pre‐specified analysis plan. In addition, the methods for this study indicated that improvement in vertigo would be assessed using the ratio of attacks before and after treatment, with a ratio of ≤ 0.8 representing improvement. However, the results only report the number of people in whom vertigo completely resolved, rather than those in whom there was some improvement. 

For Hanner 2010, no trial protocol or registration was available for assessment, but we considered it unusual to conduct a trial of Ménière's disease and only assess vertigo using a quality of life score (not using any assessment of specific vertigo symptoms). In addition, hearing was assessed as part of this study, but the outcome was not reported fully. 

#### Other potential sources of bias

We rated this domain as low risk for Kitahara 2016. For Hanner 2010, we rated this domain at unclear risk of bias, as there was very limited detail on the conduct of the study, and we had some concerns over the diagnostic criteria used for Ménière's disease, as described above. 

### Effects of interventions

See: Table 1; Table 2; Table 3

#### 1. Abundant water intake compared to no treatment

##### 1.1. Improvement in vertigo

For this outcome we included any data that were reported as a dichotomous (binary) outcome ‐ i.e. classifying participants as having improved or not improved. 

###### 1.1.1. Improvement in global score

Global improvement in vertigo was not assessed (taking account of the frequency, severity or intensity and duration of symptoms). 

###### 1.1.2. Improvement in frequency

Kitahara 2016 assessed improvement in vertigo frequency, by recording the number of participants in whom vertigo symptoms had completely resolved. 

####### 1.1.2.1. 3 to < 6 months and 6 to ≤ 12 months

This outcome was not assessed during these time periods. 

####### 1.1.2.2.  > 12 months

The study Kitahara 2016 did not assess 'any improvement' in vertigo ‐ only complete resolution was reported (having no vertigo attacks for a period of six months, i.e. between 18 and 24 months of follow‐up). Complete resolution of vertigo was more common in the group who received abundant water than the control group, with a risk ratio (RR) of 1.50 (95% confidence interval (CI) 1.18 to 1.91; 1 study; 140 participants; very low‐certainty evidence; Analysis 1.1). 

##### 1.2. Change in vertigo

For this outcome we included any continuous data ‐ where the change in vertigo was measured on a continuous scale (such as with a numerical scoring system, or the actual number of vertigo episodes experienced in a given time period). However, this outcome was not reported. 

##### 1.3. Serious adverse events

No serious adverse events were reported. It is unclear whether this is because they did not occur, or because the authors did not monitor and report adverse events.  

##### 1.4. Disease‐specific health‐related quality of life

This outcome was not reported. 

##### 1.5. Change in hearing

Improvement in hearing was assessed by Kitahara 2016, considering the number of participants in whom hearing improved by at least 10 dB over the course of the study.

###### 1.1.2.1. 3 to < 6 months and 6 to ≤ 12 months

This outcome was not assessed during these time periods. 

###### 1.1.2.2. > 12 months

More participants in the 'abundant water' group experienced an improvement in hearing, with a risk ratio of 5.00 (95% CI 2.03 to 12.31; 1 study; 140 participants; low‐certainty evidence; Analysis 1.2). 

##### 1.6. Change in tinnitus

This outcome was not reported. 

##### 1.7. Other adverse effects

No adverse effects were reported. As above, it is unclear whether this is because they did not occur, or because the authors did not monitor and report adverse effects. 

We also aimed to assess how many participants discontinued the intervention. These data were not available from the report, although the authors did specify the number of people who were excluded from analysis because they had been "<75% compliant with the rules for their group". Four out of 74 participants were excluded from the control group (5.4%), and 5 out of 75 (6.7%) were excluded from the group who were allocated to abundant water intake (RR 1.32, 95% CI 0.37 to 4.72; 1 study; 149 participants; very low‐certainty evidence; Analysis 1.3). 

#### 2. Sleeping in darkness compared to no treatment

##### 2.1. Improvement in vertigo

###### 2.1.1. Improvement in global score

Global improvement in vertigo was not assessed (taking account of the frequency, severity or intensity and duration of symptoms). 

###### 2.1.2. Improvement in frequency

Kitahara 2016 assessed improvement in vertigo frequency, by recording the number of participants in whom vertigo symptoms had completely resolved. 

####### 2.1.2.1. 3 to < 6 months and 6 to ≤ 12 months

This outcome was not assessed during these time periods. 

####### 2.1.2.2. > 12 months

The study Kitahara 2016 did not assess 'any improvement' in vertigo ‐ only complete resolution was reported. Complete resolution of vertigo was more common in the group who slept in darkness than the control group, with a risk ratio of 1.47 (95% CI 1.15 to 1.89; 1 study; 130 participants; very low‐certainty evidence; Analysis 2.1).

##### 2.2. Change in vertigo

This outcome was not reported. 

##### 2.3. Serious adverse events

No serious adverse events were reported. It is unclear whether this is because they did not occur, or because the authors did not monitor and report adverse events. 

##### 2.4. Disease‐specific health‐related quality of life

This outcome was not reported. 

##### 2.5. Change in hearing

Improvement in hearing was assessed by Kitahara 2016, considering the number of participants in whom hearing improved by at least 10 dB over the course of the study.

###### 2.1.2.1. 3 to < 6 months and 6 to ≤ 12 months

This outcome was not assessed during these time periods. 

###### 2.1.2.2. > 12 months

More participants in the 'sleeping in darkness' group experienced an improvement in hearing, with a risk ratio of 4.43 (95% CI 1.76 to 11.16; 1 study; 130 participants; low‐certainty evidence; Analysis 2.2).

##### 2.6. Change in tinnitus

This outcome was not reported. 

##### 2.7. Other adverse effects

No adverse effects were reported. As above, it is unclear whether this is because they did not occur, or because the authors did not monitor and report adverse effects. 

As described above, data on the number of participants who discontinued the intervention were not available from the report. However, the authors did specify the number of people who were excluded from analysis because they had been "<75% compliant with the rules for their group". Four out of 74 (5.4%) participants were excluded from the control group, and 14 out of 74 (18.9%) were excluded from the group who were allocated to sleeping in darkness (RR 3.50, 95% CI 1.21 to 10.14; 1 study; 148 participants; very low‐certainty evidence; Analysis 2.3). 

#### 3. Specially processed cereals compared to placebo

##### 3.1. Improvement in vertigo

This outcome was not reported. 

##### 3.2. Change in vertigo

This outcome was not reported. 

##### 3.3. Serious adverse events

No serious adverse events were reported. It is unclear whether this is because they did not occur, or because the authors did not monitor and report adverse events. 

##### 3.4. Disease‐specific health‐related quality of life

This was assessed by Hanner 2010 using the AAO‐HNS 1995 Functional Level Scale, which ranges from a score of 1 to 6, with higher scores representing worse quality of life. 

###### 3.4.1. 3 to < 6 months 

Those receiving specially processed cereals had, on average, an improvement in their quality of life as compared to those receiving placebo, with a mean difference of ‐1.35 points, but the evidence was very uncertain (95% CI ‐2.08 to ‐0.62; 1 study; 51 participants; very low‐certainty evidence; Analysis 3.1). 

###### 3.4.2. 6 to ≤ 12 months and > 12 months

This outcome was not assessed during these time periods. 

##### 3.5. Change in hearing

No numerical data were reported for this outcome. The authors state that pure tone audiometry was used to assess hearing, and that "No consistent effect in the otoneurological examinations could be demonstrated in response to the dietary treatment, and the PTA levels were unaffected in the majority of patients."

##### 3.6. Change in tinnitus

This outcome was not reported. 

##### 3.7. Other adverse effects

No adverse effects were reported. As above, it is unclear whether this is because they did not occur, or because the authors did not monitor and report adverse effects. 

No data were reported regarding the number of people who discontinued the intervention. Outcomes were reported for all participants, but it is unclear whether this is simply because an intention‐to‐treat analysis was conducted, or whether all participants continued their intervention as allocated throughout the trial. 

## Discussion

### Summary of main results

We identified one study that assessed abundant water intake or sleeping in darkness compared to no intervention. Data from 223 participants were included. Although the proportion of people who experience improvement in their vertigo may be increased by sleeping in darkness or abundant water intake, the evidence was very uncertain. No data were identified that considered a change in vertigo (as a continuous variable), or serious adverse events. Sleeping in darkness or abundant water intake may increase the number of people who experience an improvement in their hearing, however other outcomes were not assessed (tinnitus, disease‐specific health‐related quality of life and other adverse effects). The evidence on discontinuation of treatment was also very uncertain for both interventions. However, the risk of being excluded from the study due to poor adherence to the intervention was similar between the abundant water intake and the control group, but was higher in those who were allocated to sleeping in darkness. 

We also identified one study of 51 participants that assessed the use of specially processed cereals. This study did not report on any of our primary outcome measures (improvement in vertigo, change in vertigo or serious adverse events). Specially processed cereals may result in an improvement in disease‐specific health‐related quality of life, but the evidence is very uncertain. No other outcomes were reported in this study (such as tinnitus, hearing or other adverse effects). 

### Overall completeness and applicability of evidence

There was a dearth of evidence for this review, despite lifestyle and dietary changes being commonly recommended as first‐line treatments for Ménière's disease. In particular, we identified no studies that considered the effects of reducing salt or caffeine. These interventions are commonly recommended as part of first‐line therapy for Ménière's disease (Basura 2020). We only identified two studies, and they considered different interventions ‐ therefore we were unable to carry out any meta‐analysis. All the evidence we found was of very low or low certainty, showing that we are unsure of the effects of the interventions, and future research may change the effect estimates a great deal. 

This review was conducted as part of a suite considering different interventions for Ménière's disease. A number of issues were identified as affecting the completeness and applicability of the evidence in all the reviews in this suite. These have been described in the companion review on systemic pharmacological interventions for Ménière's disease (Webster 2021b), and are replicated here, as they relate to this review:

1. There is a paucity of evidence about all of these interventions, despite some of them being in common use for Ménière’s disease. All the evidence we found was of very low or low certainty, showing that we are unsure of the effects of the interventions, and future research may change the effect estimates a great deal.
2. We were unable to carry out many meta‐analyses. There were often differences in the actual outcomes assessed in the study, therefore we were unable to pool the data to achieve a more precise estimate of any effect. Study authors also often used different ways of measuring the same outcome, which prevented data from being combined. For example, vertigo was assessed with either a global score, or a frequency score, which could not be combined.
3. Certain outcomes were only assessed by some included studies. Some studies did not assess the impact of the disease on quality of life or tinnitus at all. Potential adverse effects of the interventions were also poorly reported or simply not assessed.
4. We noted that unvalidated rating scales were commonly used in the studies included, particularly when looking at the global impact of treatments for vertigo. When such scales are used, it is difficult to know if they are accurately assessing the outcome, and also what size of change on this scale represents a meaningful difference in the outcome (the minimally important difference). 
5. Finally, studies often failed to report clearly what treatments participants received before joining the trial, what maintenance treatment they continued on during the trial, and whether they received any additional treatments over the course of the trial. The impact of these additional treatments may be considerable, particularly for those studies with longer‐term follow‐up. Without knowing the background details of study participants (for example, the duration of their Ménière's disease, or what treatments they have tried in the past) it is difficult to identify the groups of people who may benefit from these treatments. 

### Quality of the evidence

We used the GRADE approach to assess the certainty of the evidence in this review. The evidence identified was all low‐ or very low‐certainty, meaning that we are uncertain about the actual effect of these interventions for all of our outcomes. The main issues that affected the certainty of the evidence were the domains of study limitations and imprecision. The different domains addressed by GRADE are considered in more detail below.

#### Study limitations/risk of bias

Both studies included in this review had at least some concerns regarding the potential for bias in the study design, conduct or reporting. Kitahara 2016 was an open‐label study, with concerns over performance and detection bias. We had concerns over the potential for selective reporting bias with Hanner 2010, and we also rated several domains at unclear risk of bias. 

#### Inconsistency

We did not conduct any meta‐analyses for this review, therefore we did not reduce the certainty of evidence due to inconsistency.

#### Indirectness

We rated down for indirectness if the outcome measured was different from that specified in our protocol. This was the case for the study Kitahara 2016, where the authors reported complete resolution of vertigo, rather than 'any improvement' in vertigo. 

#### Imprecision

All of the included studies are very small and we were unable to carry out any meta‐analyses. Therefore, the total sample size for each of our outcomes of interest was small, reducing the certainty of the evidence. For some outcomes the resulting confidence intervals for the effect size were also extremely wide ‐ meaning that there was uncertainty over whether the intervention was beneficial or harmful. This further impacted on the certainty of the evidence. 

The GRADE approach involves rating down the certainty of the evidence if the threshold for an *important* difference is crossed. For example, if the confidence interval for a result includes the possibility of an important benefit of treatment and the possibility of a trivial difference then we would reduce the certainty of the evidence by one level. If the confidence interval includes the possibility of *both* an important benefit and an important harm then the certainty would be reduced further. Therefore, it is important to agree on thresholds for this rating, i.e. where is the threshold, or cut‐point, between a trivial difference and a small, but important benefit or harm for each outcome? This question is difficult to answer, and requires input from people with balance disorders. As part of this review process, one of the author team (KW) joined some discussion groups for people with balance disorders, to try and obtain their views on quantifying an important and meaningful difference in treatment outcomes. However, the main theme that emerged from these discussions was that people were unable to give a specific threshold for each outcome. Instead, individuals tended to weigh up a variety of different factors when determining this threshold. The invasiveness and burden of taking the treatment would be taken into account, as well as potential side effects and the severity of their symptoms at that time. The GRADE working group would likely refer to this as a "fully contextualised approach", accounting for all aspects of the specific intervention in order to set thresholds for benefit (Zeng 2021). For this review we adopted a "minimally contextualised approach" and rated imprecision for each outcome according to specific, defined thresholds (as described in Methods). However, if the thresholds used are inappropriate then this may affect the certainty of the evidence (by a maximum of one level). 

#### Other considerations

We did not rate down the certainty of the evidence for other reasons. Publication bias is usually assessed as part of this domain. Although we are aware that this is an issue with many systematic reviews, we did not find strong indications of publication bias with this review. 

### Potential biases in the review process

We planned to use the Cochrane Pregnancy and Childbirth Trustworthiness Tool to assess the included studies. We had intended to exclude any study where there were concerns (as identified with this tool) from the main analyses. However, as described above, we were unable to determine whether most of the included studies would pass the screening tool, either due to a lack of reporting in the original articles, or because we were unable to contact the authors to resolve any issues. If these studies were subsequently found to have genuine concerns over research integrity then this would further undermine our confidence in the findings of the review. However, as the evidence for these interventions is all low‐ or very low‐certainty, we considered that this would not greatly impact the findings of the review. 

### Agreements and disagreements with other studies or reviews

We identified one previous Cochrane Review that considered similar interventions (Hussain 2018). However, this review contained no included studies. This is an identical finding to our review, where we did not identify any evidence on salt, caffeine or alcohol reduction in Ménière's disease. 

A clinical practice guideline, Basura 2020, identified one RCT that compared salt restriction to medical therapies for Ménière's disease (Acharya 2017), but as no placebo arm was included in this study it has not been included in our review. 

## Authors' conclusions

**Implications for practice**

There are very few randomised controlled trials (RCTs) that consider the use of lifestyle or dietary interventions for Ménière's disease. We identified a small amount of information regarding abundant water intake, sleeping in darkness or the consumption of specially processed cereals. However, the evidence available is all low‐ or very low‐certainty, therefore we cannot be sure if these interventions have any beneficial effects, nor whether they may be associated with any harm. 

**Implications for research**

**Further research on lifestyle and dietary interventions for Ménière's disease**

There are some specific challenges associated with conducting RCTs that consider lifestyle and dietary modifications (Hébert 2016). These interventions may be complex, requiring a collection of lifestyle or dietary changes for their full effect. Consequently, adherence to such interventions requires commitment from trial participants ‐ perhaps considerably more than trials of a one‐off surgical procedure, or a daily medication. Substantial dropout may be anticipated in these circumstances, leaving trials at risk of attrition bias, and underpowered to detect an effect. Furthermore, some effects of lifestyle and dietary interventions may be modest, requiring very large studies to identify any possible benefits. RCTs are costly to conduct, and it may be difficult to obtain funding for such a trial. 

Numerous confounders may cause difficulties in assessing the impact of these interventions. Dietary intake, lifestyle and behaviours of specific individuals may vary considerably at the baseline of the trial. The effects of any intervention in a specific subgroup of participants may then be masked by the mixed population included in the study. Reduction in salt intake, for example, may have different effects in those with a higher baseline intake of salt than those with relatively modest salt intake at the start of a trial. 

It is often impossible to blind participants to their allocated intervention in trials of lifestyle or dietary intervention. Typically, these interventions involve multiple modifications to diet, or specific lifestyle interventions, and it is difficult to establish a suitably blinded 'placebo' arm. Performance bias will therefore be a risk with these types of studies.

Finally, studies of lifestyle and dietary interventions may require assessment of additional outcomes when compared to trials of other interventions. Many of these interventions may be burdensome or time‐consuming to undertake, and the acceptability of this to people with Ménière's disease must also be considered. 

Even if lifestyle or dietary interventions were of only modest benefit for Ménière's disease, as these interventions may be relatively inexpensive (or free) to implement, the potential for impact is considerable. Therefore further research in this area is clearly warranted. However, it may be that evidence for these interventions will need to be established through study designs other than RCTs. The authors of future systematic reviews may need to consider data from non‐randomised studies, in order to establish whether there is any evidence for these interventions. 

**Further research on other interventions for Ménière's disease**

This review was conducted as part of a suite regarding a number of different interventions for Ménière's disease. Many of the conclusions below are relevant to all of these reviews and are replicated across the suite.

The lack of high‐certainty, RCT evidence suggests that well‐conducted studies with larger numbers of participants are required to appropriately assess the efficacy (and potential harms) of these interventions. However, there also needs to be more clarity on which outcomes studies should assess, when and how to assess them. Vertigo is a notoriously difficult symptom to assess, and there is great variety in the methods used to record and report this symptom in the studies we have identified. 

There is a clear need for consensus on which outcomes are important to people with Ménière’s disease, so that future studies can be designed with this in mind. Development of a core outcome set would be preferable as a guide for future trials. We understand that development of a core outcome set for Ménière's disease was underway, with a project registered on the COMET website (https://www.comet-initiative.org/Studies/Details/818), but we have been unable to identify any results of this project, or ascertain whether it is ongoing. If a core outcome set is developed, this should include details on the recommended methods used to measure outcomes, ensuring that these are validated, reliable tools. Monitoring and reporting of adverse effects should be considered a routine part of any study, and should always occur ‐ this is inconsistent at present. Agreement is also needed on the appropriate times at which outcomes should be measured to adequately assess the different interventions.

Any decisions about which outcomes to measure, how to measure them and when to measure them must be made with input from people with Ménière’s disease, to ensure that the outcomes reported by trialists (and future systematic reviews) are relevant to those with the disease. 

For those considering development of a core outcome set, we would highlight that the use of the dichotomous outcome 'improvement' or 'no improvement' of vertigo may cause difficulties when interpreting the results. Individuals with Ménière's disease typically experience fluctuations in disease severity over time. Furthermore, they may have enrolled in a clinical trial at a time when their symptoms were severe. Therefore there is likely to be a natural tendency to improve over time, even for those who do not receive an intervention. The high rate of improvement in those who receive no treatment means that smaller studies are likely to be underpowered to detect a true effect of treatment. Ideally, agreement should be reached on what constitutes a *meaningful improvement* in vertigo symptoms, rather than simply considering any improvement as a positive outcome. 

Trialists should also be clear about the treatments that participants received before entry to the trial, throughout the trial, and the need for additional treatment during the course of the trial. People with Ménière's disease need to be able to understand whether interventions work in all people with the disease, or whether they might work best during certain phases of the disease ‐ perhaps as a first‐line therapy, or for people in whom other treatments have failed. 

## History

Protocol first published: Issue 1, 2022

## Appendices

### Appendix 1. AAO‐HNS definition of Ménière's disease

Definite Ménière's disease:

- Two or more spontaneous episodes of vertigo, each lasting 20 minutes to 12 hours.
- Audiometrically documented low‐ to medium‐frequency sensorineural hearing loss in one ear, defining the affected ear on at least one occasion before, during or after one of the episodes of vertigo.
- Fluctuating aural symptoms (hearing, tinnitus or fullness) in the affected ear.
- Not better accounted for by another vestibular diagnosis.

Probable Ménière's disease: 

- Two or more spontaneous episodes of vertigo, each lasting 20 minutes to 24 hours.
- Fluctuating aural symptoms (hearing, tinnitus or fullness) in the affected ear.
- Not better accounted for by another vestibular diagnosis.

Taken from Lopez‐Escamez 2015.

### Appendix 2. Search strategy

This search strategy has been designed to identify all relevant studies for a suite of reviews on various interventions for Ménière's disease. 

**Table:**

**CENTRAL (CRS) **	**Cochrane ENT Register (CRS) **	**MEDLINE (Ovid) **
1 MESH DESCRIPTOR Endolymphatic Hydrops EXPLODE ALL AND CENTRAL:TARGET 2 meniere*):AB,EH,KW,KY,MC,MH,TI,TO AND CENTRAL:TARGET 3 (endolymphatic near hydrops):AB,EH,KW,KY,MC,MH,TI,TO AND CENTRAL:TARGET 4 (labyrinth* near hydrops):AB,EH,KW,KY,MC,MH,TI,TO AND CENTRAL:TARGET 5 (labyrinth* near syndrome):AB,EH,KW,KY,MC,MH,TI,TO AND CENTRAL:TARGET 6 (aural near vertigo):AB,EH,KW,KY,MC,MH,TI,TO AND CENTRAL:TARGET 7 (labyrinth* near vertigo):AB,EH,KW,KY,MC,MH,TI,TO AND CENTRAL:TARGET 8 (cochlea near hydrops):AB,EH,KW,KY,MC,MH,TI,TO AND CENTRAL:TARGET 9 (vestibular near hydrops):AB,EH,KW,KY,MC,MH,TI,TO AND CENTRAL:TARGET 10 #1 OR #2 OR #3 OR #4 OR #5 OR #6 OR #7 OR #8 OR #9 AND CENTRAL:TARGET	1 MESH DESCRIPTOR Endolymphatic Hydrops EXPLODE ALL AND INREGISTER 2 (meniere*):AB,EH,KW,KY,MC,MH,TI,TO AND INREGISTER 3 (endolymphatic near hydrops):AB,EH,KW,KY,MC,MH,TI,TO AND INREGISTER 4 (labyrinth* near hydrops):AB,EH,KW,KY,MC,MH,TI,TO AND INREGISTER 5 (labyrinth* near syndrome):AB,EH,KW,KY,MC,MH,TI,TO AND INREGISTER 6 (aural near vertigo):AB,EH,KW,KY,MC,MH,TI,TO AND INREGISTER 7 (labyrinth* near vertigo):AB,EH,KW,KY,MC,MH,TI,TO AND INREGISTER 8 (cochlea near hydrops):AB,EH,KW,KY,MC,MH,TI,TO AND INREGISTER 9 (vestibular near hydrops):AB,EH,KW,KY,MC,MH,TI,TO AND INREGISTER 10 #1 OR #2 OR #3 OR #4 OR #5 OR #6 OR #7 OR #8 OR #9 AND INREGISTER 11 INREGISTER 12 * AND CENTRAL:TARGET 13 #11 NOT #12 14 #10 AND #13	1 exp Endolymphatic Hydrops/ 2 meniere*.ab,ti. 3 (endolymphatic adj3 hydrops).ab,ti. 4 (labyrinth* adj3 hydrops).ab,ti. 5 (labyrinth* adj3 syndrome).ab,ti. 6 (aural adj3 vertigo).ab,ti. 7 (labyrinth* adj3 vertigo).ab,ti. 8 (cochlea adj3 hydrops).ab,ti. 9 (vestibular adj3 hydrops).ab,ti. 10 1 or 2 or 3 or 4 or 5 or 6 or 7 or 8 or 9 11 randomized controlled trial.pt. 12 controlled clinical trial.pt. 13 randomized.ab. 14 placebo.ab. 15 drug therapy.fs. 16 randomly.ab. 17 trial.ab. 18 groups.ab. 19 11 or 12 or 13 or 14 or 15 or 16 or 17 or 18 20 exp animals/ not humans.sh. 21 19 not 20 22 10 and 21
**Embase (Ovid) **	**Web of Science Core Collection (Web of Knowledge) **	**Trial Registries **
1 exp Meniere disease/ 2 meniere*.ab,ti. 3 (endolymphatic adj3 hydrops).ab,ti. 4 (labyrinth* adj3 hydrops).ab,ti. 5 (labyrinth* adj3 syndrome).ab,ti. 6 (aural adj3 vertigo).ab,ti. 7 (labyrinth* adj3 vertigo).ab,ti. 8 (cochlea adj3 hydrops).ab,ti. 9 (vestibular adj3 hydrops).ab,ti. 10 1 or 2 or 3 or 4 or 5 or 6 or 7 or 8 or 9 11 Randomized controlled trial/ 12 Controlled clinical study/ 13 Random$.ti,ab. 14 randomization/ 15 intermethod comparison/ 16 placebo.ti,ab. 17 (compare or compared or comparison).ti. 18 ((evaluated or evaluate or evaluating or assessed or assess) and (compare or compared or comparing or comparison)).ab. 19 (open adj label).ti,ab. 20 ((double or single or doubly or singly) adj (blind or blinded or blindly)).ti,ab. 21 double blind procedure/ 22 parallel group$1.ti,ab. 23 (crossover or cross over).ti,ab. 24 ((assign$ or match or matched or allocation) adj5 (alternate or group$1 or intervention$1 or patient$1 or subject$1 or participant$1)).ti,ab. 25 (assigned or allocated).ti,ab. 26 (controlled adj7 (study or design or trial)).ti,ab. 27 (volunteer or volunteers).ti,ab. 28 human experiment/ 29 trial.ti. 30 11 or 12 or 13 or 14 or 15 or 16 or 17 or 18 or 19 or 20 or 21 or 22 or 23 or 24 or 25 or 26 or 27 or 28 or 29 31 (random$ adj sampl$ adj7 ("cross section$" or questionnaire$1 or survey$ or database$1)).ti,ab. 32 comparative study/ or controlled study/ 33 randomi?ed controlled.ti,ab. 34 randomly assigned.ti,ab. 35 32 or 33 or 34 36 31 not 35 37 Cross‐sectional study/ 38 randomized controlled trial/ or controlled clinical study/ or controlled study/ 39 (randomi?ed controlled or control group$1).ti,ab. 40 38 or 39 41 37 not 40 42 (((case adj control$) and random$) not randomi?ed controlled).ti,ab. 43 (Systematic review not (trial or study)).ti. 44 (nonrandom$ not random$).ti,ab. 45 Random field$.ti,ab. 46 (random cluster adj3 sampl$).ti,ab. 47 (review.ab. and review.pt.) not trial.ti. 48 we searched.ab. 49 review.ti. or review.pt. 50 48 and 49 51 update review.ab. 52 (databases adj4 searched).ab. 53 (rat or rats or mouse or mice or swine or porcine or murine or sheep or lambs or pigs or piglets or rabbit or rabbits or cat or cats or dog or dogs or cattle or bovine or monkey or monkeys or trout or marmoset$1).ti. and animal experiment/ 54 36 or 41 or 42 or 43 or 44 or 45 or 46 or 47 or 50 or 51 or 52 55 30 not 54 56 10 and 55	# 12 #11 AND #10 Indexes=SCI‐EXPANDED, SSCI, A&HCI, CPCI‐S, CPCI‐SSH, BKCI‐S, BKCI‐SSH, ESCI, CCR‐EXPANDED, IC Timespan=All years # 11 TOPIC: ((randomised OR randomized OR randomisation OR randomisation OR placebo* OR (random* AND (allocat* OR assign*) ) OR (blind* AND (single OR double OR treble OR triple) ))) Indexes=SCI‐EXPANDED, SSCI, A&HCI, CPCI‐S, CPCI‐SSH, BKCI‐S, BKCI‐SSH, ESCI, CCR‐EXPANDED, IC Timespan=All years # 10 #9 OR #8 OR #7 OR #6 OR #5 OR #4 OR #3 OR #2 OR #1 Indexes=SCI‐EXPANDED, SSCI, A&HCI, CPCI‐S, CPCI‐SSH, BKCI‐S, BKCI‐SSH, ESCI, CCR‐EXPANDED, IC Timespan=All years # 9 TOPIC: (vestibular NEAR/3 hydrops) Indexes=SCI‐EXPANDED, SSCI, A&HCI, CPCI‐S, CPCI‐SSH, BKCI‐S, BKCI‐SSH, ESCI, CCR‐EXPANDED, IC Timespan=All years # 8 TOPIC: (cochlea NEAR/3 hydrops) Indexes=SCI‐EXPANDED, SSCI, A&HCI, CPCI‐S, CPCI‐SSH, BKCI‐S, BKCI‐SSH, ESCI, CCR‐EXPANDED, IC Timespan=All years # 7 TOPIC: (labyrinth* NEAR/3 vertigo) Indexes=SCI‐EXPANDED, SSCI, A&HCI, CPCI‐S, CPCI‐SSH, BKCI‐S, BKCI‐SSH, ESCI, CCR‐EXPANDED, IC Timespan=All years # 6 TOPIC: (labyrinth* adj3 vertigo) Indexes=SCI‐EXPANDED, SSCI, A&HCI, CPCI‐S, CPCI‐SSH, BKCI‐S, BKCI‐SSH, ESCI, CCR‐EXPANDED, IC Timespan=All years # 5 TOPIC: (aural NEAR/3 vertigo) Indexes=SCI‐EXPANDED, SSCI, A&HCI, CPCI‐S, CPCI‐SSH, BKCI‐S, BKCI‐SSH, ESCI, CCR‐EXPANDED, IC Timespan=All years # 4 TOPIC: (labyrinth* NEAR/3 syndrome) Indexes=SCI‐EXPANDED, SSCI, A&HCI, CPCI‐S, CPCI‐SSH, BKCI‐S, BKCI‐SSH, ESCI, CCR‐EXPANDED, IC Timespan=All years # 3 TOPIC: (labyrinth* NEAR/3 hydrops) Indexes=SCI‐EXPANDED, SSCI, A&HCI, CPCI‐S, CPCI‐SSH, BKCI‐S, BKCI‐SSH, ESCI, CCR‐EXPANDED, IC Timespan=All years # 2 TOPIC: (endolymphatic NEAR/3 hydrops) Indexes=SCI‐EXPANDED, SSCI, A&HCI, CPCI‐S, CPCI‐SSH, BKCI‐S, BKCI‐SSH, ESCI, CCR‐EXPANDED, IC Timespan=All years # 1 TOPIC: (meniere*) Indexes=SCI‐EXPANDED, SSCI, A&HCI, CPCI‐S, CPCI‐SSH, BKCI‐S, BKCI‐SSH, ESCI, CCR‐EXPANDED, IC Timespan=All years	Clinicaltrials.gov menieres or meniere or meniere's | Interventional Studies ICTRP Meniere*

The date restrictions applied to the September 2022 update searches were as follows:

**Table:**

** **	**September 2022 update**
**CENTRAL**	15/09/2021_TO_14/09/2022:CRSINCENTRAL AND CENTRAL:TARGET
**ENT register**	No new records added to register since search was run
**MEDLINE**	23 limit 22 to ed=20210915‐20220914 24 limit 22 to dt=20210915‐20220914 25 23 or 24
**Embase**	57 limit 56 to dd=20210915‐20220914
**Web of Science**	Timespan: 2021‐09‐15 to 2022‐09‐14 (Index Date)
**Clinicaltrials.gov**	First posted from 09/15/2021 to 09/14/2022
**ICTRP**	Date of registration after 15/09/2021
**Google Scholar**	Year: 2021 or 2022

### Appendix 3. Trustworthiness Screening Tool

This screening tool has been developed by Cochrane Pregnancy and Childbirth. It includes a set of predefined criteria to select studies that, based on available information, are deemed to be sufficiently trustworthy to be included in the analysis. These criteria are:

**Research governance**

- Are there any retraction notices or expressions of concern listed on the Retraction Watch Database relating to this study?
- Was the study prospectively registered (for those studies published after 2010)? If not, was there a plausible reason?
- When requested, did the trial authors provide/share the protocol and/or ethics approval letter?
- Did the trial authors engage in communication with the Cochrane Review authors within the agreed timelines?
- Did the trial authors provide IPD data upon request? If not, was there a plausible reason?

**Baseline characteristics**

- Is the study free from characteristics of the study participants that appear too similar (e.g. distribution of the mean (SD) excessively narrow or excessively wide, as noted by Carlisle 2017)?

**Feasibility**

- Is the study free from characteristics that could be implausible? (e.g. large numbers of women with a rare condition (such as severe cholestasis in pregnancy) recruited within 12 months);
- In cases with (close to) zero losses to follow‐up, is there a plausible explanation?

**Results**

- Is the study free from results that could be implausible? (e.g. massive risk reduction for main outcomes with small sample size)?
- Do the numbers randomised to each group suggest that adequate randomisation methods were used (e.g. is the study free from issues such as unexpectedly even numbers of women ‘randomised’ including a mismatch between the numbers and the methods, if the authors say ‘no blocking was used’ but still end up with equal numbers, or if the authors say they used ‘blocks of 4’ but the final numbers differ by 6)?

Studies assessed as being potentially ‘high risk’ will be not be included in the review. Where a study is classified as ‘high risk’ for one or more of the above criteria we will attempt to contact the study authors to address any possible lack of information/concerns. If adequate information remains unavailable, the study will remain in ‘awaiting classification’ and the reasons and communications with the author (or lack of) described in detail.

The process is described in full in Figure 2. 

## Data and analyses

**Comparison 1:** Abundant water intake versus no treatment

Outcome or subgroup title	No. of studies	No. of participants	Statistical method	Effect size
1.1 Improvement in frequency of vertigo (complete resolution) at > 12 months	1	140	Risk Ratio (M‐H, Random, 95% CI)	1.50 [1.18, 1.91]
1.2 Change in hearing: improvement in hearing at > 12 months	1	140	Risk Ratio (M‐H, Random, 95% CI)	5.00 [2.03, 12.31]
1.3 Other adverse effects: exclusion from follow‐up	1	144	Risk Ratio (M‐H, Random, 95% CI)	1.32 [0.37, 4.72]

**Comparison 2:** Sleeping in darkness versus no treatment

Outcome or subgroup title	No. of studies	No. of participants	Statistical method	Effect size
2.1 Improvement in frequency of vertigo (complete resolution) at > 12 months	1	130	Risk Ratio (M‐H, Random, 95% CI)	1.47 [1.15, 1.89]
2.2 Change in hearing: improvement in hearing at > 12 months	1	130	Risk Ratio (M‐H, Random, 95% CI)	4.43 [1.76, 11.16]
2.3 Other adverse effects: exclusion from follow‐up	1	148	Risk Ratio (M‐H, Random, 95% CI)	3.50 [1.21, 10.14]

**Comparison 3:** Specially processed cereals versus placebo

Outcome or subgroup title	No. of studies	No. of participants	Statistical method	Effect size
3.1 Disease‐specific health‐related quality of life (Functional Level Scale)	1	51	Mean Difference (IV, Random, 95% CI)	‐1.35 [‐2.08, ‐0.62]

## Characteristics of studies

### Characteristics of included studies [ordered by study ID]

**Table:** Hanner 2010

***Study characteristics***		
Methods	Parallel‐group, double‐blind, placebo‐controlled study. Duration of treatment (and follow‐up) 3 months. This study included an optional, additional open‐arm 3‐month prospective cohort study for those in the placebo group to have the active treatment, but the results of this treatment phase are not included in this review.	
Participants	**Setting: ** Single‐centre study of participants who were being treated at the Sahlgrenska University Hospital in Sweden. Study dates are not reported. **Sample size:** **Number randomised:** 51 participants **Number completed:** 51 participants **Participant baseline characteristics** **Age: ** Specially processed cereal (SPC) flakes group: mean 57 years (range 12 to 79) Control group: mean 56 years (range 41 to 72) **Gender: ** SPC flakes group: 14 males (51.9%): 13 females (48.1%) Control group: 14 males (58.3%): 10 females (41.7%) **Probable/definite Ménière's disease: ** All definite disease (inclusion criterion). However, there is some uncertainty over the exact criteria used to diagnose participants with Ménière's disease. **Duration of disease: ** SPC flakes group: mean 12 years (range 1 to 37 years) Control group: mean 14 years (range 1 to 30 years) **Attack frequency at baseline: ** Not reported **Hearing loss at baseline:** Not reported **Measure of tinnitus at baseline: ** Not reported **Number of participants with bilateral disease: ** Not reported **Inclusion criteria:** Male and female participants; aged 12 to 80 years; definite Ménière's disease; undergoing treatment at Sahlgrenska University Hospital, Sweden **Exclusion criteria:** Not specified **Diagnostic criteria used for Ménière's disease:** The authors state that participants had "definite Ménière's disease", and cite the AAO‐HNS 1995 guidelines elsewhere in the article. However, the criteria for diagnosis are not explicitly stated. The authors provided us with some further information, stating that "The diagnostic criteria established by Swedish and European were used to diagnose the patients. Furthermore, most of these patients had been followed by Per Hanner for several years. This means that the knowledge of the patient as well of the disease development/response to commonly used medication was good."	
Interventions	**Intervention (n = 27 randomised, n = 27 completed)** Specially processed cereal flakes. The SPC flakes were taken as a dietary supplement for a 3‐month long experimental period. The cereals were consumed together with milk, twice daily as a dietary supplement, at a dose of 1 g/kg body weight/day. **Comparator (n = 24 randomised, n = 24 completed)** Placebo flakes (cereals without anti‐secretory factor inducing activity). The placebo flakes were taken as a dietary supplement for a 3‐month long experimental period. The cereals were consumed together with milk twice daily as a dietary supplement, at a dose of 1 g/kg body weight/day. **Background interventions administered to all participants** None; no changes were made to the participant's routine medication	
Outcomes	**Primary outcomes relevant to this review:** **Improvement in vertigo** Not reported **Change in vertigo** Not reported **Serious adverse events** Not reported **Secondary outcomes relevant to this review:** **Disease‐specific health‐related quality of life** FLS score, according to the AAO‐HNS 1995, was reported at the end of the trial (3 months) **Hearing** Assessed with pure tone audiometry and speech audiometry. Only reported narratively, no numeric data are presented. **Tinnitus ** Not reported **Other adverse effects** Not reported	
Funding sources	SPC flakes and control cereals were a gift from Lantmännen AS‐Faktor AB. Financial support was provided by the Göteborg Medical Society, the Swedish State under the LUA agreement (grant # 7157), Sahlgrenska University Hospital (grant # 8303) and the Magnus Bergvall Foundation.	
Declarations of interest	The authors report no conflicts of interest	
Notes	**Research integrity checklist:** No retractions/expressions of concern were identified Limited information on baseline characteristics of participants was reported. However, there are no concerns regarding excessive similarity based on the details we can assess. No loss to follow‐up was reported A very marked effect size is seen in the FLS score changes, despite the small sample size No concerns over the adequacy of randomisation	
***Risk of bias***		
**Bias**	**Authors' judgement**	**Support for judgement**
Random sequence generation (selection bias)	Low risk	Quote: "The patients were randomized by lottery to receive either active treatment with SPC‐flakes or placebo". Comment: simple drawing of lots.
Allocation concealment (selection bias)	Unclear risk	Comment: insufficient details regarding allocation process to be confident about this. Authors do state that the "the study code [was] kept secret at the Department of Audiology, Sahlgrenska University Hospital, until all data had been processed".
Blinding of participants and personnel (performance bias) All outcomes	Low risk	Quote: "Neither the physician nor the patients knew which package contained SPC‐flakes or placebo." (E. Jennische, personal communication) Comment: placebo cereals used. Unlikely that participants could have identified the active from placebo versions of the cereals. Study personnel were also blinded to group allocation.
Blinding of outcome assessment (detection bias) All outcomes	Low risk	Comment: outcomes were assessed by (blinded) participants themselves.
Incomplete outcome data (attrition bias) All outcomes	Low risk	Comment: no missing outcome data.
Selective reporting (reporting bias)	High risk	Comment: very limited outcome data, no assessment of vertigo episodes other than through a quality of life score, and these would be considered key outcomes for a study of Ménière's disease. Audiometry outcomes were assessed but not reported in sufficient detail to be included in a meta‐analysis. No protocol was available for comparison.
Other bias	Unclear risk	Comment: insufficient detail on the methods and conduct of study, and baseline details regarding participants to judge. It is unclear whether the AAO‐HNS 1995 guidelines for diagnosis of Meniere's disease were used.

**Table:** Kitahara 2016

***Study characteristics***		
Methods	4‐arm, parallel‐group, open‐label RCT with 24 months total duration of treatment and follow‐up. 3 arms are relevant to this review, and the data below pertain to these arms. The 4th arm (ventilation tubes) is relevant for, and included in, a separate review in this suite (Webster 2021a).	
Participants	**Setting: ** Single‐centre study. Recruitment took place in the vertigo and dizziness department of Osaka Rosai Hospital in Japan from January 2010 until December 2013. **Sample size:** **Number randomised:** 223 participants included in the 3 arms relevant to this review (297 total in the trial) **Number completed:** 200 participants included in the 3 arms relevant to this review (263 total in the trial) **Participant baseline characteristics** **Age:** Abundant water intake group: mean 46.7 years (SD 13.7) Sleeping in darkness group: mean 49.7 years (SD 11.9) Control group: mean 49.7 years (SD 16.5) **Gender:** Abundant water intake group: 28 male (40%): 42 female (60%) Sleeping in darkness group: 22 male (36.7%): 38 female (63.3%) Control group: 25 males (35.7%): 45 females (64.3%) **Probable/definite Ménière's disease: ** All definite disease **Duration of disease: ** Abundant water intake group: mean 32.1 months (SD 22.2) Sleeping in darkness group: mean 33.8 months (SD 23.2) Control group: mean 30.3 months (SD 22.7) **Attack frequency at baseline: ** Abundant water intake group: mean 1.7 attacks per month (SD 1) Sleeping in darkness group: mean 1.7 attacks per month (SD 1.1) Control group: mean 1.6 attacks per month (SD 1) **Hearing loss at baseline:** Abundant water intake group: mean 48.7 dB hearing level (SD 14.9) Sleeping in darkness group: mean 49.1 dB hearing level (SD 15.1) Control group: mean 47.3 dB hearing level (SD 13.4) **Measure of tinnitus at baseline: ** Not reported **Number of participants with bilateral disease: ** None (table of demographics reports laterality of disease for each participant; no participant is listed as having bilateral disease, therefore we assume all were unilateral) **Inclusion criteria:** Definite MD according to the AAO‐HNS 1995 criteria. Age ≥ 20 years. Eligible patients were those in whom 3 to 6 months of "basically fixed forms of medical treatment" had produced insufficient benefit (i.e. recurrent vertigo attacks and/or no improvement in sensorineural hearing loss) and for whom surgical treatment would otherwise be considered. Medical treatments included diuretics, betahistine, diphenidol, dimenhydrinate and diazepam. No further information on doses or treatments in each group was provided. **Exclusion criteria:** Cardiac disease; renal dysfunction; middle ear problems; nyctophobia (those who cannot sleep in darkness) Participants were excluded from the trial if they did not adhere to their treatment (< 75% adherence) **Criteria for diagnosis of Ménière's disease:** Definite MD according to the AAO‐HNS 1995 criteria	
Interventions	**Intervention A (n = 75 randomised, n = 70 completed)** Abundant water intake plus medication. Participants were required to have abundant water intake: 35 mL/kg/day with a self‐check diary of water volume/day. They also continued traditional medication as per the control group. **Intervention B (n = 74 randomised, n = 60 completed)** Sleeping in darkness plus medication. Participants were given advice to sleep in darkness to maintain the hormonal circadian rhythm, defined as lying in bed in an unlit room less than 0.1 lux for 6 to 7 hours per night with a self‐check diary using an illuminometer (Panasonic Corp., Osaka, Japan). They also continued traditional medication as per the control group. **Comparator (n = 74 randomised, n = 70 completed)** Medication only. Participants continued to receive traditional oral medications: "Medical treatments were fixed basically including diuretics, betahistine, diphenidol, dimenhydrinate, and diazepam, all considered effective for persistent symptoms of Meniere’s disease". **Background interventions administered to all participants** As described above, all 3 groups received oral medication	
Outcomes	**Primary outcomes relevant to this review:** **Improvement in vertigo** Improvement of vertigo was assessed using the control of vertigo scale, according to the AAO‐HNS 1995, with a score of < 0.8 being regarded as improvement. However, the article only reports the proportion of participants who had complete resolution of vertigo. **Change in vertigo** Not reported **Serious adverse events** Not reported; trial registration document does list "all cause mortality" as the primary outcome measure **Secondary outcomes relevant to this review:** **Disease‐specific health‐related quality of life** Not assessed or reported **Hearing** Assessed with pure tone audiometry with an average of four frequencies (0.5 kHz, 1 kHz, 2 kHz and 4 kHz). Reported as a dichotomous outcome: improved/not improved, with a threshold of a decrease of 10 dB representing improvement. **Tinnitus ** Not assessed or reported **Other adverse effects** Not assessed or reported **Other outcomes reported in the study:** Blood results for stress (pAVP and cortisol) Questionnaires measuring psychological factors (SDS and SRS‐18)	
Funding sources	This study was supported in part by JSPS KAKENHI Grant (2016‐2018) and by Grant‐in‐Aid for Research on Intractable Vestibular Disorder from Japan AMED (2015‐2017). The funders above had no role in study design, data collection and analysis, decision to publish or preparation of the manuscript.	
Declarations of interest	The authors have declared that no competing interests exist	
Notes	**Research integrity checklist:** No retractions/expressions of concern were identified Baseline characteristics of the groups were not excessively similar Dropouts and withdrawals were described The effect size for all of the interventions is considerably larger than for the control group, despite all participants in the trial receiving standard treatment for Ménière's disease, and the control group achieving considerable improvement over the course of the trial Blocked randomisation was used, accounting for similar number of participants allocated to each group	
***Risk of bias***		
**Bias**	**Authors' judgement**	**Support for judgement**
Random sequence generation (selection bias)	Low risk	Quote: "The remaining 297 definite Meniere's patients were randomly assigned through computer‐generated block randomization organized by the clinical study section at Osaka Rosai Hospital to one of four treatment groups." Comment: computer‐generated block randomisation by a third party.
Allocation concealment (selection bias)	Low risk	Quote: "The remaining 297 definite Meniere's patients were randomly assigned through computer‐generated block randomization organized by the clinical study section at Osaka Rosai Hospital to one of four treatment groups." Comment: computer‐generated block randomisation and allocation by a third party.
Blinding of participants and personnel (performance bias) All outcomes	High risk	Comment: this was an open‐label study. Participants and study personnel were aware of group allocation.
Blinding of outcome assessment (detection bias) All outcomes	High risk	Comment: this was an open‐label study. Participants were aware of their group allocation, and outcomes are reported by (unblinded) participants and study personnel.
Incomplete outcome data (attrition bias) All outcomes	Low risk	Comment: the number of dropouts was low (< 10%) and balanced across the groups. This should have been insufficient to cause a significant change in the estimated effect size.
Selective reporting (reporting bias)	High risk	Quote: "A ratio of pre/post‐treatment values of ≤0.8 was regarded as "better", or ≥1.2 as "worse" and the remainder as no change". Comment: vertigo improvement was stated to be assessed as better/worse/unchanged in the methods. However, the authors only report the number of participants in whom vertigo resolved completely over the course of the study. Although the study was prospectively registered with clinicaltrials.gov, the only outcome listed here is "all cause mortality". This seems a surprising choice of primary outcome measure, and is also not reported in the publication.
Other bias	Low risk	Comment: no other concerns were identified.

**AAO‐HNS:** American Academy Otolaryngology ‐ Head and Neck Surgery; **FLS:** (AAO‐HNS) Functional Level Scale; **MD:** Ménière's disease; **pAVP:** plasma arginine vasopressin; **SD:** standard deviation; **SDS:** Self‐rating Depression Scale; **SPC:** specially processed cereal; **SRS‐18:** Stress Response Scale‐18

### Characteristics of excluded studies [ordered by study ID]

**Table:**

Study	Reason for exclusion
Cui 2022	The intervention (vestibular rehabilitation) was not relevant for this review
Faag 2017	This study included a mixed population of participants with different vestibular disorders; no data are reported that are specific to those with Ménière's disease
Garcia 2013	This RCT of vestibular rehabilitation with virtual reality only followed participants up for a maximum of 6 weeks
Hong 2011	This intervention (Chinese medicine) was not relevant for the review
Hussain 2018	This is a systematic review with no included studies
Ingvardsen 2016	This was a cross‐over trial with only 2 months of follow‐up for each phase; data for the first phase of the trial could not be extracted
Lee 2019	The population for this study was not appropriate ‐ participants were required to have chronic dizziness, but not necessarily Ménière's disease
Leong 2013	This was a cross‐over trial with a follow‐up period of 3 months; data from the first phase of the trial could not be extracted from the publication
Liu 2020	This RCT included an insufficient duration of follow‐up (only 1 month)
NCT05328895	The intervention was not appropriate; we did not consider transcranial auricular nerve stimulation to be a lifestyle intervention
NCT05355610	This is not a RCT
Richards 1971	This is a narrative review article and not an RCT
Shin 2017	The population is not appropriate for this review ‐ a mixed group of people with dizziness were to be included, according to the study protocol
Sun 2014	The intervention is not appropriate for this review
Yardley 2006	The population included were individuals who self‐identified as having Ménière's disease (recruited through a survey of the Ménière's Society); therefore no diagnostic criteria were used to establish the diagnosis of Ménière's disease as part of the study
Zhong 2008	The intervention is not appropriate for this review

RCT: randomised controlled trial

### Characteristics of ongoing studies [ordered by study ID]

**Table:** IRCT20201102049238N1

Study name	The effect of Nigella sativa on Meniere's disease
Methods	Single‐blind (participant‐blinded) RCT with 3 months duration of treatment and follow‐up
Participants	Target sample size: 40 participants **Inclusion criteria:** Participants with definite Ménière's disease; no age limit **Exclusion criteria:** Previous cardiac, hepatic, renal, psychiatric or hypertensive disease
Interventions	**Intervention** 1 g Nigella sativa oil daily **Control** 1 g placebo daily
Outcomes	Improvement in hearing loss (measured with audiometry) Improvement in tinnitus (measured with the THI) Improvement in vertigo (measured with the DHI)
Starting date	Anticipated start date: 6 April 2021 Anticipated end of enrolment: 22 October 2021
Contact information	Zahra Rabbani z‐rabbani@student.tums.ac.ir
Notes	—

**Table:** IRCT20210716051908N1

Study name	Evaluation of the effect of vitamin D supplementation in reducing vertigo attacks and severity in Meniere’s patients, a randomized double‐blind clinical trial with placebo‐controlled
Methods	Randomised, double‐blind, placebo‐controlled trial
Participants	Adult participants with a definitive diagnosis of Ménière's disease according to the guidelines of the American Academy of Otolaryngology (AAO‐HNS) Aged over 18 years Serum levels of 25 (OH) vitamin D3 less than 30 ng/mL and more than 10 ng/mL
Interventions	**Intervention group:** in addition to receiving betahistine, Pearl vitamin D supplement 50,000 units, one per week for 8 weeks will be prescribed **Control group:** the placebo, which is exactly the same as the vitamin D supplement in shape and colour, will be delivered once a week for 8 weeks. (It is unclear whether participants in the control group will also receive betahistine).
Outcomes	Severity of dizziness (visual analogue scale) Number of dizzy episodes per month Mean duration of vertigo Frequency multiplied by duration of vertigo Mean severity of tinnitus
Starting date	October 2021
Contact information	Tehran University of Medical Sciences
Notes	This trial may have insufficient follow‐up duration for future updates of this review (as outcomes are stated to be measured at 8 weeks). However, it has been included here in case longer follow‐up is also conducted.

**DHI:** Dizziness Handicap Inventory; **RCT:** randomised controlled trial; **THI:** Tinnitus Handicap Inventory

## Differences between protocol and review

We planned to use the Trustworthiness Screening Tool from Cochrane Pregnancy and Childbirth to identify studies for inclusion in the main analysis. However, as described in Selection of studies, we did not exclude studies from the main analysis on the basis of concerns whilst using this tool. We considered that the overall certainty of the review findings (all very low‐ or low‐certainty) would not be impacted by this decision. 
